# Ultrasensitive detection of intact SARS-CoV-2 particles in complex biofluids using microfluidic affinity capture

**DOI:** 10.1126/sciadv.adh1167

**Published:** 2025-01-10

**Authors:** Daniel C. Rabe, Adarsh Choudhury, Dasol Lee, Evelyn G. Luciani, Uyen K. Ho, Alex E. Clark, Jeffrey E. Glasgow, Sara Veiga, William A. Michaud, Diane Capen, Elizabeth A. Flynn, Nicola Hartmann, Aaron F. Garretson, Alona Muzikansky, Marcia B. Goldberg, Douglas S. Kwon, Xu Yu, Aaron F. Carlin, Yves Theriault, James A. Wells, Jochen K. Lennerz, Peggy S. Lai, Sayed Ali Rabi, Anh N. Hoang, Genevieve M. Boland, Shannon L. Stott

**Affiliations:** ^1^ Krantz Family Center for Cancer Research, Massachusetts General Hospital, Boston, MA, USA.; ^2^ Center for Engineering in Medicine and Surgery, Massachusetts General Hospital, Harvard Medical School, Boston, MA, USA.; ^3^ Department of Medicine, Massachusetts General Hospital, Harvard Medical School, Boston, MA, USA.; ^4^ Broad Institute of Harvard and Massachusetts Institute of Technology, Cambridge, MA, USA.; ^5^ Departments of Pathology and Medicine, School of Medicine, University of California, San Diego, La Jolla, CA, USA.; ^6^ Department of Bioengineering and Therapeutic Sciences, University of California, San Francisco, San Francisco, CA, USA.; ^7^ Department of Surgery, Massachusetts General Hospital, Harvard Medical School, Boston, MA, USA.; ^8^ Microscopy Core of the Program in Membrane Biology, Massachusetts General Hospital, Boston, MA, USA.; ^9^ Massachusetts General Hospital Biostatistics, Harvard Medical School, Boston, MA, USA.; ^10^ Infectious Diseases, Massachusetts General Hospital, Harvard Medical School, Boston, MA, USA.; ^11^ Department of Microbiology, Harvard Medical School, Boston, MA, USA.; ^12^ Department of Immunology and Infectious Diseases, Harvard T.H. Chan School of Public Health, Boston, MA, USA.; ^13^ Ragon Institute of MGH, MIT, and Harvard, Cambridge, MA, USA.; ^14^ Qualcomm Institute, University of California, San Diego, La Jolla, CA, USA.; ^15^ Department of Pathology, Massachusetts General Hospital, Harvard Medical School, Boston, MA, USA.

## Abstract

Measuring virus in biofluids is complicated by confounding biomolecules coisolated with viral nucleic acids. To address this, we developed an affinity-based microfluidic device for specific capture of intact severe acute respiratory syndrome coronavirus 2 (SARS-CoV-2). Our approach used an engineered angiotensin-converting enzyme 2 to capture intact virus from plasma and other complex biofluids. Our device leverages a staggered herringbone pattern, nanoparticle surface coating, and processing conditions to achieve detection of as few as 3 viral copies per milliliter. We further validated our microfluidic assay on 103 plasma, 36 saliva, and 29 stool samples collected from unique patients with COVID-19, showing SARS-CoV-2 detection in 72% of plasma samples. Longitudinal monitoring in the plasma revealed our device’s capacity for ultrasensitive detection of active viral infections over time. Our technology can be adapted to target other viruses using relevant cell entry molecules for affinity capture. This versatility underscores the potential for widespread application in viral load monitoring and disease management.

## Teaser

Microfluidics can be used to isolate intact viral particles from a variety of biofluids potentially informing infectivity.

## INTRODUCTION

### Viral load detection in plasma and other biofluids

The ability to isolate and detect whole virus from complex biofluids would enable a greater understanding for how severe acute respiratory syndrome coronavirus 2 (SARS)–CoV-2 (or other viral infections) spread through the host and the provide insights into the dynamics of this journey. A quantitative measurement of whole viral particles could inform infectivity and a possible correlation between viral load and end organ damage. Measurements of viral RNA in nasopharyngeal swabs cannot ascertain whether viral RNA is shed or encapsulated in a virus. During the pandemic, many studies showed that viral RNA was present in nasopharyngeal swabs at least 1 month past initial COVID-19 positivity making unclear whether patients were still infectious or simply shedding viral RNA (*1*–*6*). Being able to distinguish between shed viral RNA and intact viral particles could help distinguish recovered patients from those with latent virus. In COVID-19 infection, there are conflicting reports on how many patients have viral RNA in their plasma, ranging from 0 to 40% (*7*). It is thought that if virus is present, it may only be traces of viral RNA, rather than intact viral particles in the blood (*8*). Yet, many studies have shown blood to be rich with cell and molecular signatures of infection and immune response. Bulk (*9*) and single-cell (*10*) RNA sequencing of peripheral blood cells shows hallmarks of SARS-CoV-2 infection, suggesting that blood and plasma may be untapped resources for viral testing. Studying additional biofluids (like saliva and stool) would further provide insight into infectivity, viral kinetics, and patient outcome. Saliva samples have been an attractive source of material to test for SARS-CoV-2 because of its proximity to the nasopharynx (*11*–*14*). Saliva has been proposed as a means of more rapid point of care screening methods (*13*, *15*–*18*). In addition, some studies have suggested that saliva samples are a better material for screening than nasopharyngeal swabs (*11*, *12*), with some further arguing saliva tests are more sensitive in detecting SARS-CoV-2 in patients that are asymptomatic or have mild cases of COVID-19 (*19*). Furthermore, high levels of SARS-CoV-2 found in wastewater studies (*20*–*25*), as well as gastrointestinal tract symptoms associated with COVID-19 (*26*–*32*), would be informative to additionally test SARS-CoV-2 levels in the stool of patients with COVID-19 during infection to provide insight into patient disease state.

The ability to isolate and detect whole virus would enable a greater understanding of the viral kinetics of viremia and viral seeding, leading to end organ damage. A quantitative measurement of whole viral particles could also help us better understand viral infectivity of biospecimens, as well as potential for transmission between individuals. While several commercial RNA extraction kits can be automated for clinical laboratory use, many molecular assays are limited by their ability to amplify extracted viral RNA from complex biofluids like plasma or whole blood due to the contamination of several polymerase chain reaction (PCR) inhibitory molecules including hemoglobin, immunoglobin G (IgG), lactoferrin, or anticoagulants (EDTA, citrate, or heparin) used for sample preservation (*33*–*36*). Despite improvements in reducing carryover of PCR-inhibitory molecules, these direct RNA extraction methods will isolate free viral RNA in addition to intact viral particles. Therefore, it is necessary to design assays that can distinguish between free viral RNA and intact viral particles in a sample.

### Microfluidic detection of viruses

Microfluidics offer the ability to have precise control over cells and particles, allowing for isolation and analyses or rare components in complex fluids via density-, size-, acoustic-, or affinity-based methods (*37*–*43*). For example, microfluidic devices have been used to isolate and detect viral components soon after they appear in an infected individual (*44*–*50*). In addition, they can be cost-effective, offer more portable or rapid methods of detection, and require very small input volumes (*51*). Following isolation of viral material, a variety of detection methods can be used to detect SARS-CoV-2 infection or immune response (*44*, *52*), including PCR or loop-mediated isothermal amplification (*53*) to detect viral RNA or DNA, enzyme-linked immunosorbent assay or immune fluorescent signal to detect viral proteins (*54*) or virus-targeting human antibodies or combinations of both antigens and antibodies (*46*, *55*), tunable resistive pulse sensing to quantify purified viral particles (*37*), or dielectric microsensors (*56*). Microfluidic devices have been used for detecting viral infection through measurement of antiviral antibodies in a variety of viral outbreaks, including Ebola (*57*), HIV (*58*), influenza (*59*–*61*), Zika (*62*–*64*), dengue (*65*, *66*), SARS-CoV-2 (*16*, *18*, *45*, *46*, *67*–*70*), and other respiratory viruses (*71*, *72*). Microfluidic chips can be designed to accommodate multiple detection stages within the same integrated device, allowing for easier use and translatability (*37*). Microfluidic devices are an attractive potential source for the development of point of care–based assays that do not require high complexity clinical laboratories and can be easier translated to areas with fewer resources (*73*).

### Severe acute respiratory syndrome coronavirus 2

COVID-19 was first reported in December 2019 in Wuhan, China (*74*, *75*) and declared a pandemic in March 2020 by the World Health Organization (*75*). Sequencing of viral RNA samples from infected individuals identified severe acute respiratory syndrome coronavirus 2 (SARS-CoV-2), which shares 50 to 88% sequence identity with other coronaviruses (*76*). Modeling of SARS-CoV-2 receptor binding domain (RBD) predicted a similar structure to SARS-CoV, suggesting angiotensin-converting enzyme 2 (ACE2) as a means of viral entry (*76*). Crystal structures of the SARS-CoV-2 RBD bound to ACE2 confirmed that it is the receptor necessary for viral entry into host cells (*77*, *78*). Mutations within the RBD of the spike protein of SARS-CoV-2, seen in different variants, can alter the ability of the virus to bind ACE2. When examining the effect of mutations of concern within the RBD of spike present in the Delta variant (B.1.617.2) using cryo–electron microscopy analysis, the T478K substitution in particular lead to increased binding potential to ACE2 (*79*). By contrast, the BA.1 subvariant of Omicron (B.1.1.529), harbors additional mutations in the RBD of spike resulting in decreased binding to ACE2 and greater immune escape (*80*–*82*).

Following a SARS-CoV-2 exposure, most patients develop symptoms within 4 to 5 days (*83*). Symptoms, ranging from mild to severe, include fever, dry cough, shortness of breath, headache, general fatigue, dizziness, vomiting, and/or diarrhea (*84*–*86*). Those at greater risk of developing more severe disease or Long Covid include patients with hypertension, diabetes mellitus, over the age of 65, obesity, chronic obstructive pulmonary disease, other respiratory diseases, cardiovascular disease, or cancer (*87*–*89*).

### Viral detection using our virus isolation chip (^virus^HB-Chip)

**Fig. 1.**
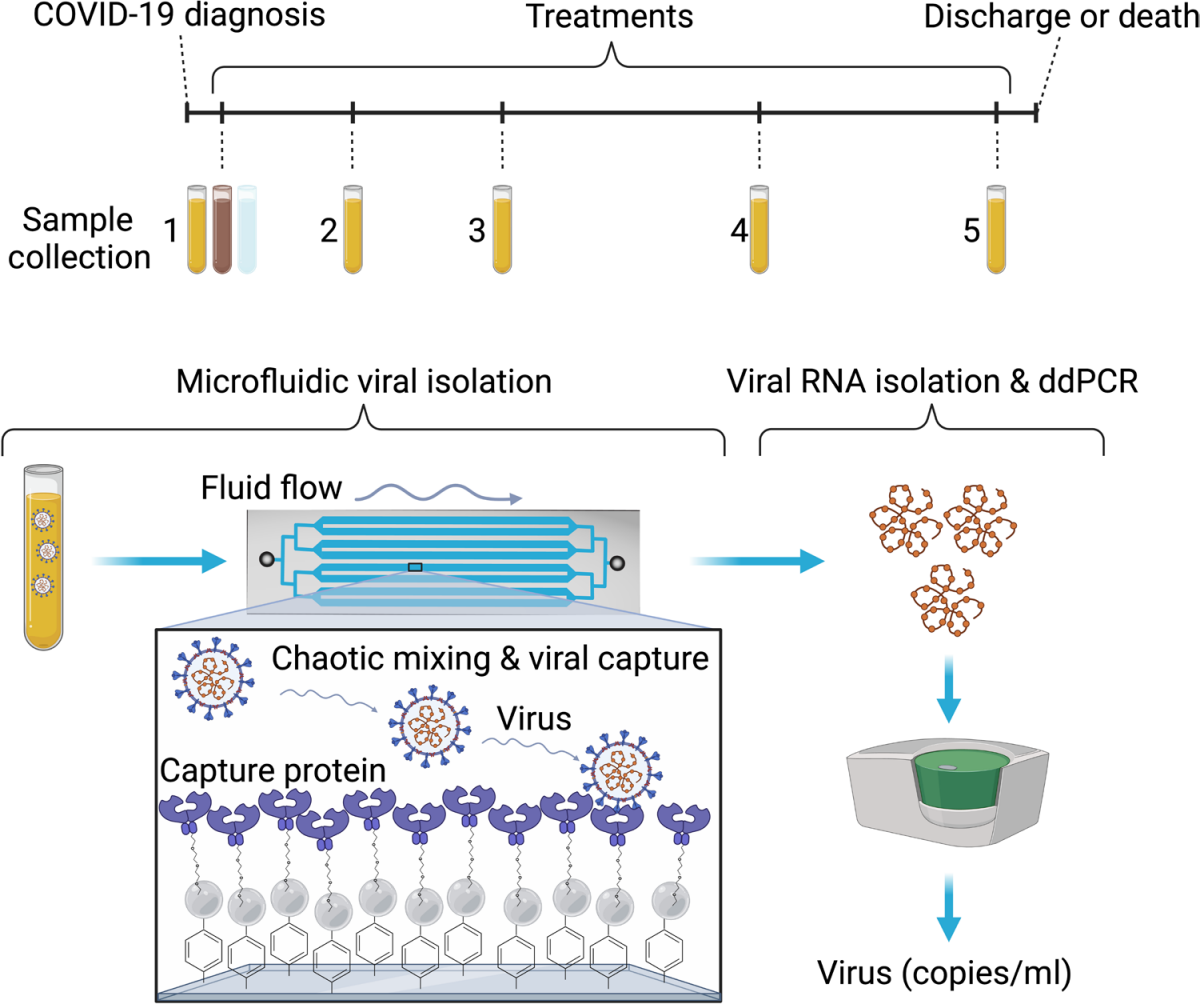
Clinical workflow and viral detection using the ^virus^HB-Chip. (**Top**) Schematic describing clinical specimen collection and timing from patients with COVID-19 after diagnosis and during treatments. We obtained individual plasma samples from 103 patients, stool from 29, and saliva from 36. We obtained serial plasma samples from 10 patients during treatment. (**Bottom**) Schematic describing microfluidic isolation of virus from patient plasma using the ^virus^HB-Chip, followed by viral RNA isolation from the chip, and subsequent viral copies per milliliter calculated using droplet digital (ddPCR). Created using BioRender.com.

In this study, we demonstrate that we created a virus isolation chip (^virus^HB-Chip) for capture of intact viral particles from a variety of patient biofluids, including plasma, stool, and saliva (Fig. 1). Traditionally, plasma is a highly complex fluid in which it is difficult to detect virus because of coisolation of many inhibitor molecules. To isolate low-abundance viral particles from plasma, we optimized a herringbone-grooved microfluidic chip (*90*, *91*) for viral particle capture. Taking an immunoaffinity capture approach, we isolated virus capsids, using an engineered version of the ACE2 receptor (ACE2^ENG^) capable of binding SARS-CoV-2 spike protein at higher rates than wild-type (WT) ACE2 (ACE2^WT^) (*92*). The engineered version of ACE2 we used in this study was designed by the Wells laboratory as a receptor trap that could be used therapeutically in patients with COVID-19. Because of the high-affinity binding that resulted from its design, it was an optimal choice for our assay to isolate intact viral particles from complex biofluids using an affinity-based capture method. Rather than using ACE2^ENG^ as a receptor trap to bind intact viral particles blocking them from viral entry, we used it to capture intact viral particles in collaboration with the Wells laboratory. We then extracted viral RNA from the chip and directly detecting viral RNA copies using Bio-Rad’s triplex reverse transcription droplet digital polymerase chain reaction (RT-ddPCR) SARS-CoV-2 assay. This ultimately resulted in an ultrasensitive method for detection of intact virus in plasma, saliva, and stool. The specificity of our device for intact SARS-CoV-2 was determined by challenging it with other respiratory viruses, as well as measuring its ability to detect variants known to display increased binding to ACE2. The sensitivity of our assay was determined using blinded limit of detection panels. Last, our assay was benchmarked in a Clinical Laboratory Improvement Amendments (CLIA) environment using clinically annotated samples, testing our ability to detect SARS-CoV-2 in plasma from 103 patients, stool from 29 patients, and saliva from 36 patients. We additionally tested plasma from 10 patients collected serially during treatment. Overall, we have shown our device can detect intact ultrarare viral particles in plasma, suggesting that it could be used to test viral load in plasma for other viral infections by quickly altering the affinity capture molecules chosen.

## RESULTS

### Using microfluidic affinity capture for viral diagnostic applications in plasma

**Fig. 2.**
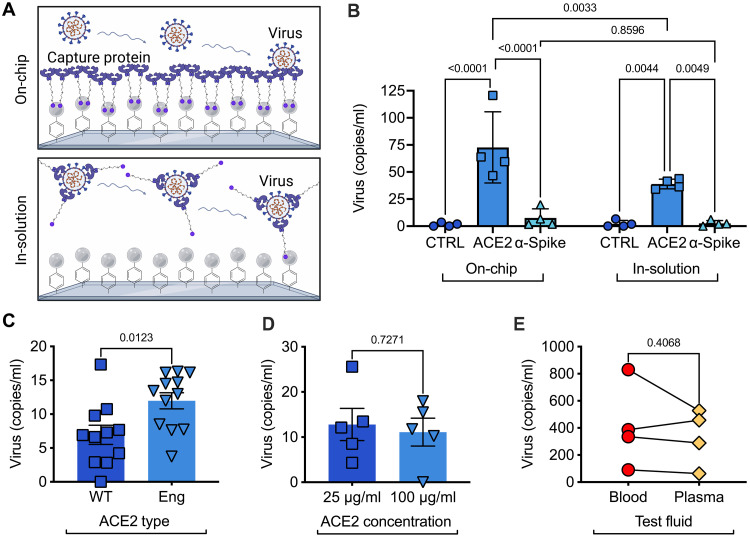
Microfluidic capture of virus using the ^virus^HB-Chip. (**A**) Schematic describing chaotic mixing of fluid as it flows across the staggered herringbone pattern of the HB-Chip. ACE2 functionalized to the surface captures SARS-CoV-2 spike protein as the fluid flows across the surface. Created using BioRender.com. (**B** to **E**) SARS-CoV-2 was diluted and spiked into healthy donor plasma or blood (E). Viral RNA was detected using ddPCR. (B) SARS-CoV-2 was captured on the ^virus^HB-Chip using either a nonspecific IgG (CTRL, dark blue circles), ACE2^WT^ (light blue squares), or an anti-spike protein antibody (aqua triangles). Capture molecules are added to the device before use (left) or incubated with the sample before being added to the device (right). (C) Capture of SARS-CoV-2 using recombinant ACE2^WT^ (dark blue squares) or ACE2^ENG^ (Eng ACE2, light blue triangles). (D) Capture of SARS-CoV-2 using a solution of 25 μg/ml (dark blue squares) or 100 μg/ml of ACE2^ENG^ (light blue triangles) added to the chip before capture. (E) Capture of virus from blood (red circles) or from a volume corrected amount of plasma (plasma-colored diamonds) using the ^virus^HB-Chip with ACE2^ENG^.

To isolate intact viral particles from blood, we targeted the SARS-CoV-2 spike (S) protein that decorates the envelope of the virus. Once captured on the ^virus^HB-Chip, we directly extracted RNA from captured viral particles in the chip and used one-step RT-ddPCR to determine the number of viral copies of SARS-CoV-2 N1 and N2 RNA as well as human ribonuclease P/MRP subunit p30 (RPP30) RNA. For this study, we explored two methods to target the spike protein: using the ACE2 protein itself (*76*) or an antibody against the spike protein. To control interactions between the virus and our selected capture moiety, we used a microfluidic approach, wherein a staggered herringbone structure was built into the surface of the device to create passive mixing (*93*), increasing analyte-surface interactions as complex biofluids flow through the device (*94*). While we had used a similar approach to enrich cancer cells and extracellular vesicles in the past (*90*, *91*, *95*), neither we nor others have attempted to use this approach to isolate viral particles. Many have used immunoaffinity strategies to isolate biological cells with microfluidics, and optimal performance is often achieved when capture molecules are preattached to the inner surface of the chip. Knowing that intact-virus isolation assays often relied on tagging the virus particle in solution (*96*), we explored whether the biophysics of these nanometer sized particles might have an impact on our isolation strategy. We first compared whether capture of virus occurred best by either (i) flowing healthy donor plasma spiked with virus through the chip with the capture molecule attached to the inner surface of the chip (Fig. 2A, On-Chip) or (ii) mixing our capture protein in solution with virus spiked into healthy donor plasma, then allowing the biotin-tagged capture molecule to bind to streptavidin beads functionalized to the inner surface of our chip (Fig. 2A, In-Solution). We additionally examined whether anti-spike antibodies or human ACE2 served as better capture molecules (Fig. 2A). UV-C (ultraviolet-C)–inactivated SARS-CoV-2 Delta was spiked into healthy donor plasma that did not contain anti-spike antibodies (fig. S1). RNA was then extracted directly from virus captured on the chip and viral copies were determined using a triplex SARS-CoV-2 N1 and N2 and human RPP30 RT-ddPCR triplex assay. By averaging the copies of N1 and N2, we could measure the number of viral particles (containing both a copy of SARS-CoV-1 N1 and N2). We then corrected for the volume of plasma (or other biofluid) processed to determine viral copies per milliliter of sample. RPP30 served as a positive control for RNA extraction. We found that placing our capture molecule directly on-chip led to increased levels of viral capture when using ACE2 (*P* = 0.0033; Fig. 2B and fig. S2). When we compared our two capture moieties (ACE2 versus αspike), we found that ACE2 had a much higher rate of viral capture in the ^virus^HB-Chip regardless of how it was used (*P* < 0.0001, On-Chip; *P* = 0.0049, In-Solution). By comparison, a nonspecific IgG showed minimal to no binding of SARS-CoV-2 in the chip (*P* < 0.0001; Fig. 2B and fig. S2). We therefore chose to further optimize our device using ACE2 functionalized to the inner surface of the ^virus^HB-Chip for viral capture.

### Determining the optimal microfluidic immunoaffinity capture agent

To further increase the capture efficiency of our ^virus^HB-Chip, we tested whether an engineered ACE2 (ACE2^ENG^) receptor trap could capture virus at a higher rate (*92*). Using a combination of computational and yeast screening, Glasgow *et al.* (*92*) designed an ACE2 receptor trap fused to a human immunoglobulin crystallizable fragment (Fc) domain that bound the RBD of spike protein and neutralized binding to target cells (median inhibitory concentration = 10 to 100 ng/ml). Because of its ability to bind SARS-CoV-2 at an even higher rate than WT ACE2 (ACE2^WT^), we wanted to test its ability to capture SARS-CoV-2 in the ^virus^HB-Chip. Using inactivated virus spiked into healthy donor plasma, we found that ACE2^ENG^ showed an ~2-fold increase in viral capture in our ^virus^HB (*P* = 0.0123; Fig. 2C and fig. S3) in comparison to ACE2^WT^. To find the optimal capture protein concentration to functionalize the ^virus^HB-Chip, we tested a range of concentrations from 25 μg of the ACE2^ENG^ to 100 μg/ml. We found that higher amounts of ACE2^ENG^, did not increase viral binding, suggesting that 25 μg/ml is sufficient to fully saturate the chip with capture molecules (Fig. 2D).

We next tested whether our viral isolation strategy was compatible with whole blood. Success with whole blood would eliminate the need for an extra processing step (i.e., plasma separation) in a clinical environment, while also demonstrating potential utility for at-home testing. We also anticipated the level of intact SARS-CoV-2 particles to be quite low in patient samples and knew that the act of plasma separation could destroy some of these particles. Whole blood from COVID-19 (−) individuals was spiked with inactivated SARS-CoV-2. Half of each sample prepared was separated to be processed as is. The remaining half of the spiked blood sample was processed for plasma isolation. Four unique donors were used for these data. We found that when corrected for the percentage of plasma volume, there was no difference in capture efficiency of SARS-CoV-2 from whole blood or plasma (*P* = 0.4068), suggesting that our device could be adapted for settings where a faster test in whole blood is needed (Fig. 2E).

### Optimization and automation of viral RNA extraction

Knowing that particle size, surface charge, and deformability can affect microfluidic performance, we further optimized each processing step to increase our detection sensitivity. Once captured, viral RNA extraction methods can have a significant impact on yield. For all methods tested, viral RNA was extracted on-chip, because of the increased shear forces that promote virus lysis. Using low dead-volume syringes, rather than traditional syringes with high dead volume (fig. S4A) as well as vertical orientation of the syringes (fig. S4B) increased our ability to detect virus bound to our chip twofold each (*P* = 0.0174, *P* = 0.0091, respectively). Using a bead-based automated RNA extraction method, we found ~50-fold higher levels of viral RNA bound to our chip compared to manual column-based RNA extraction (*P* = 0.01; fig. S4C). Using a one-step RT-ddPCR detection resulted in a >300-fold higher level of viral detection compared to a two-step method (*P* = 0.0101; fig. S4D). Using a synthetic blocking solution, rather than a 3% bovine serum albumin block in phosphate-buffered saline (PBS), containing 0.05% Tween 20, led to a 12-fold increase in detection of virus (*P* = 0.1702; fig. S4E). In addition, we created a method for automating the lysis step in the chips to make our assay more translatable to clinical laboratories needing to process many samples (fig. S5 and movie S1).

### Capture of intact viral particles using the ^virus^HB-Chip

**Fig. 3.**
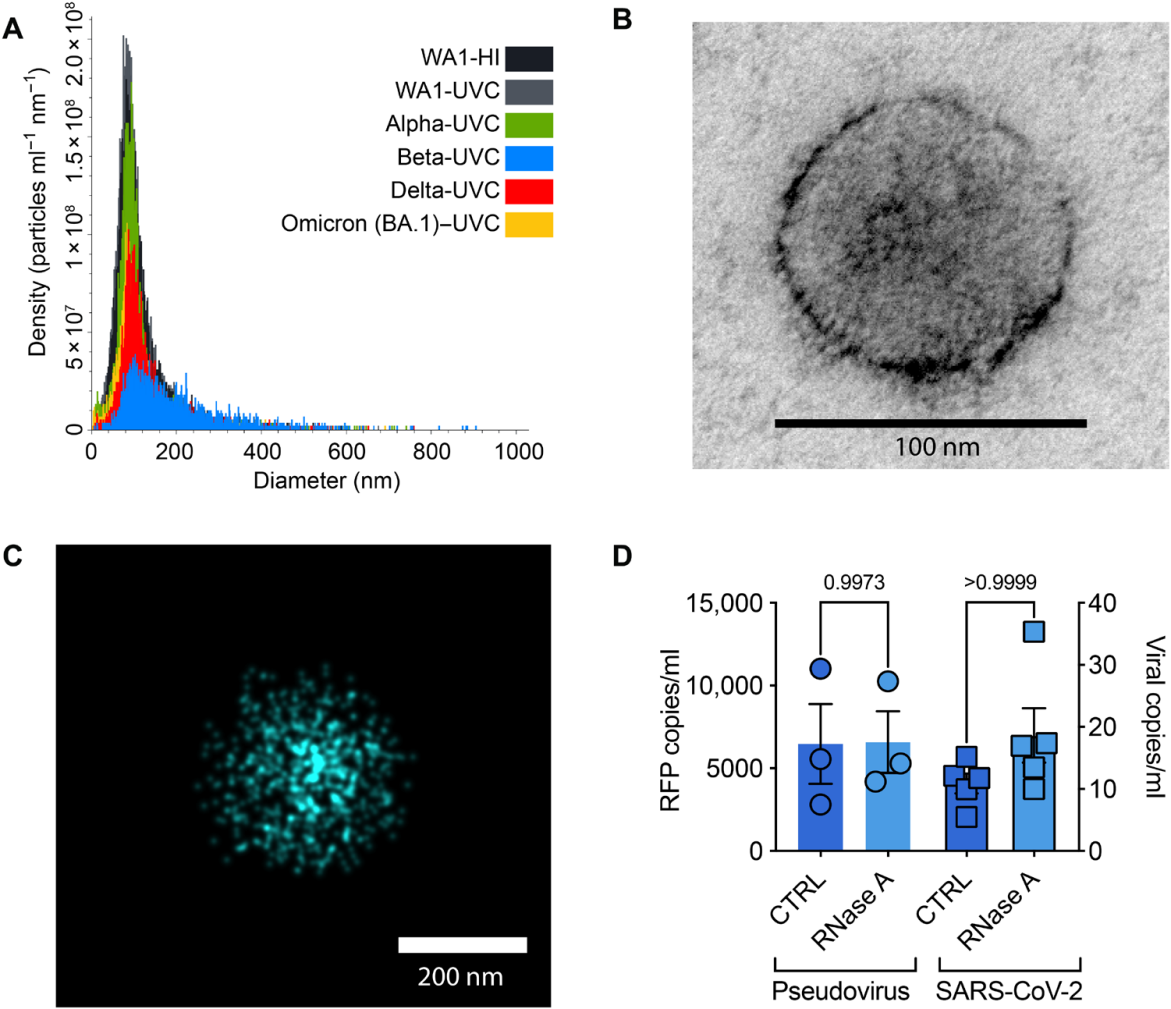
Capture of intact virus by the ^virus^HB-Chip. (**A** to **C**) Inactivated SARS-CoV-2 in solution was subjected to NTA (A), TEM (B), and dSTORM (C) to determine its biophysical characteristics before further analysis. (A) Density of viral particles and diameter of different strains of heat-inactivated (HI) or UV-C–inactivated (UVC) SARS-CoV-2 determined by NTA. (B) Representative TEM image captured of inactivated SARS-CoV-2 (Delta variant, UV-C inactivated). (C) Representative image captured of inactivated SARS-CoV-2 (Delta variant, UVC inactivated) captured on the ONI EV-profiler chip using ACE2^ENG^, stained with anti-spike antibody, and imaged using dSTORM with a TIRF illumination angle using the Nanoimager from Oxford Nanoimaging (ONI). (**D**) Either SARS-CoV-2 spike protein pseudotyped virus (Pseudovirus) or SARS-CoV-2 (Delta variant, UV-C inactivated) was captured on the ^virus^HB-Chip and detected by ddPCR. Before RNA extraction, captured virus [Pseudovirus, circles (left); SARS-CoV-2, squares (right)] were subjected to either PBS (CTRL, dark blue) or RNase A (light blue) for 30 min at 37**°**C. *P* values were calculated using a *t* test.

To determine whether our ^virus^HB-Chip was capturing intact viral particles, rather than viral RNA binding to the interior surface of the chip, we took several different strategies. We first measured the concentrations and median diameter of inactivated viral particles in solution using nanoparticle tracking analysis (NTA). We did not observe a shift in median size between the different strains or inactivation methods (Fig. 3A and table S1). Different aliquots showed different particle concentrations that corresponded to ddPCR measurements of viral preparations, suggesting that viral RNA was contained within viral particles and not shed during viral production. To further examine whether our viral particles were intact, we used transmission electron microscopy (TEM) to examine the viral corona. Inactivated viral preparations were fixed, plated on a Formvar/carbon-coated nickel grid, and subjected to TEM. TEM confirmed that we had intact viral capsids with a similar size of viral capsids determined by NTA (Fig. 3B). While we observed some viral particles larger than 200 nm in all detection modalities, the vast majority had a mean diameter near 100 nm, measured by NTA. We further showed intact viral capsids captured on the extracellular vesicle (EV)-profiler chip using ACE2^ENG^ and direct stochastic optical reconstruction microscopy (dSTORM) of the SARS-CoV-2 spike glycoprotein using a total internal reflective fluorescence (TIRF) illumination angle (Fig. 3C). The viral particles analyzed and using the EV-profiler kit tended to more robustly detect the larger viral particles of ~200 nm. While our chip capture strategy contains a rigorous washing step, and we have demonstrated previously that there are very low levels of nonspecific binding, we wanted to further explore whether it was possible we were detecting free viral RNA that had simply bound to the chip nonspecifically. First, we treated viral samples captured on the ^virus^HB-Chip to either a 30-min treatment at 37°C with ribonuclease A (RNase A) or PBS. Following RNase A treatment, we saw no loss in captured virus by ddPCR using either a SARS-CoV-2 pseudotyped lentivirus or inactivated SARS-CoV-2 (Fig. 3D). This suggests that viral RNA detected by ddPCR was protected from RNase A degradation because it was contained in the interior of the viral particle.

### Effects of SARS-CoV-2 variants on ^virus^HB-Chip capture efficiency

**Fig. 4.**
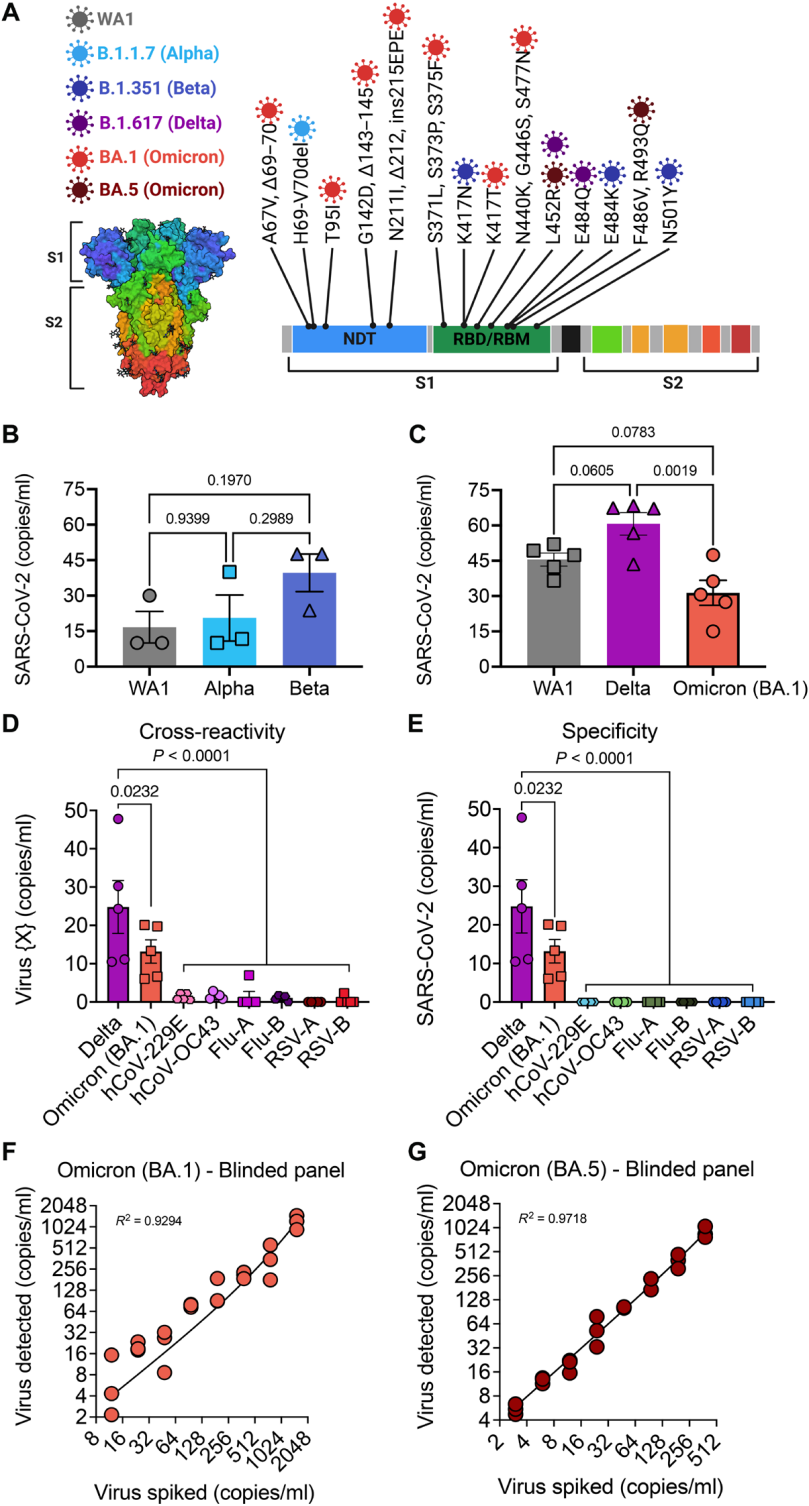
Specificity and sensitivity of the ^virus^HB-Chip. (**A**) Left: A diagram of SARS-CoV-2 spike protein is shown for PDB file (6VXX). Different domains of the protein are color coded corresponding to the domains shown in a linear sequence to the right. Right: Diagram of mutations of interest in the spike protein of different variants of SARS-CoV-2. Mutations are color coded for each of the major variants of interest. Created using BioRender.com. (**B** and **C**) Similar amounts of virus were spiked into healthy donor plasma separately for each variant. WA1 (gray circles), Alpha variant (aqua squares), Beta variant (light blue triangles), Delta variant (fuchsia triangles), or Omicron BA.1 variant (orange circles) were detected by ddPCR after being captured on the ^virus^HB chip. (**D** and **E**) Delta or Omicron (BA.1) variants of SARS-CoV-2 or hCoV-229E, hCoV-OC43, Flu-A, Flu-B, RSV-A, or RSV-B were separately spiked into healthy donor plasma. Detected viral copies were normalized for amount loaded. (D) SARS-CoV-2 detection was measured by ddPCR for all samples. (E) Detection of other respiratory viruses bound to the ^virus^HB-Chip was measured using viral specific primers and probes for each different virus. *P* values were calculated using a one-way analysis of variance (ANOVA) with correction for multiple testing. (**F**) Copies of Omicron (BA.1) detected are graphed on the *y* axis versus spiked copies calculated from a blinded dilution series created by the RADx-rad Discoveries and Data Coordinating Center (DCC) on the *x* axis (*N* = 3 per dilution). (**G**) Copies of Omicron (BA.5) detected are graphed on the *y* axis versus spiked copies calculated from a blinded dilution series created by the DCC on the *x* axis (*N* = 3 per dilution).

After optimizing the chip for viral capture, we wanted to determine whether ACE2^ENG^ was able to capture different variants of SARS-CoV-2 as efficiently, as each had unique mutations in the spike glycoprotein. Figure 4A summarizes the mutations of interest seen in the Alpha, Beta, Gamma, Delta, Omicron (BA.1), and Omicron (BA.5) variants. As can be seen, the BA.1 subvariant of Omicron displayed a much larger number of mutations in the spike glycoprotein than the others. Heat-inactivated viral samples showed a lower rate of binding to the ^virus^HB-Chip (fig. S6A) as well as a corresponding damage to the protein corona of the virus detected by TEM (fig. S6, B and C). We therefore proceeded to use viral samples inactivated with UV-C alone. When we compared capture efficiency of the Alpha and Beta variants to the WA1 variant, we found that Beta displayed a higher capture efficiency compared to WA1, which would be expected given that some of the mutations it had gained would give it a higher binding capacity to ACE2 (Fig. 4B). When we compared Delta and Omicron to WA1, we found a similar trend with higher binding rates of Delta, and lower binding rates of the BA.1 subvariant (Fig. 4C). While exact levels of viral capture are slightly affected by the ability of different variants of SARS-CoV-2, our microfluidic assay is capable of detecting all variants.

### Cross-reactivity and specificity of the ^virus^HB-Chip for SARS-CoV-2

To test the cross-reactivity of our device for capture of other respiratory viruses, we tested healthy donor plasma that was spiked with different inactivated viruses. Specifically, we tested coronaviruses (hCoV-229E and hCoV-OC43), different strains of influenza [H3N2 influenza A (Flu-A) and influenza B (Flu-B)], and other respiratory viruses (RSV-A or RSV-B), with SARS-CoV-2 Delta and Omicron (BA.1) as controls. Each sample was flown through a separate chip functionalized with ACE2^ENG^. Each sample was tested with a ddPCR probe that matched the virus flown through the chip. We found that the other viruses showed little cross-reactivity to the chip compared to SARS-CoV-2 strain Delta or Omicron (BA.1) (Fig. 4D). To test specificity of our PCR probes, none of the non–SARS-CoV-2 viral samples was detected when we performed PCR with the SARS-CoV-2 ddPCR probes (Fig. 4E).

### Limit of detection of the ^virus^HB-Chip

As part of the RADx-Rad initiative to increase rigor and reproducibility of funded works (including this study), a Discoveries and Data Coordinating Center (DCC) was created. A part of RADx-rad DCC’s objectives was to provide standardized testing materials for validation of studies. In addition to inactivated virus provided for our work, the DCC provided us with blinded limit of detection panels based off our previously measured limit of detection (fig. S7, A and B). These samples were resuspended in healthy donor plasma and processed through the ^virus^HB-Chip. After processing, we determined viral copies per milliliter of each sample and were then unblinded to the spiked concentrations, including three negative samples. The device detected SARS-CoV-2 in blinded samples as low as 12 copies per milliliter for Omicron BA.1 with a detection efficiency of 65% detection of expected copies across all samples (Fig. 4F) and 3 copies per milliliter for Omicron BA.5 with a detection efficiency of 208% detection of expected copies across all samples (Fig. 4G) and no nonspecific detection in negative samples. This demonstrated that our platform captures particles in plasma at concentrations as low as 3 viral particles per milliliter. Because our assay is able to detect SARS-CoV-2 at or below 12 copies per milliliter across multiple variants, we believe our assay can perform agnostic to the variant present in a clinic sample (Fig. 4, F and G, and fig. S7).

### Viral detection in patients

After optimizing our platform and validating the limit of detection using RADx-Rad blinded samples (3 viral particles per milliliter), we used the ^virus^HB-Chip to measure the number of intact viral particles in a SARS-CoV-2 patient cohort. All samples were collected from patients presenting to the emergency department who were enrolled in a clinical study approved by the Mass General Brigham Human Research Committee, the governing institutional review board (IRB) at Massachusetts General Hospital (*97*). Each patient had a verified COVID-19 diagnosis using quantitative PCR (qPCR) from nasopharyngeal swabs in a CLIA laboratory. While samples were collected at multiple time points, most samples tested were collected within 2 to 3 days of the COVID-19 diagnosis. We selected this time, as it was anticipated that viral levels were likely to be highest in biofluids early during infection.

#### Detection rate of intact SARS-CoV-2 virus in patient plasma

**Fig. 5.**
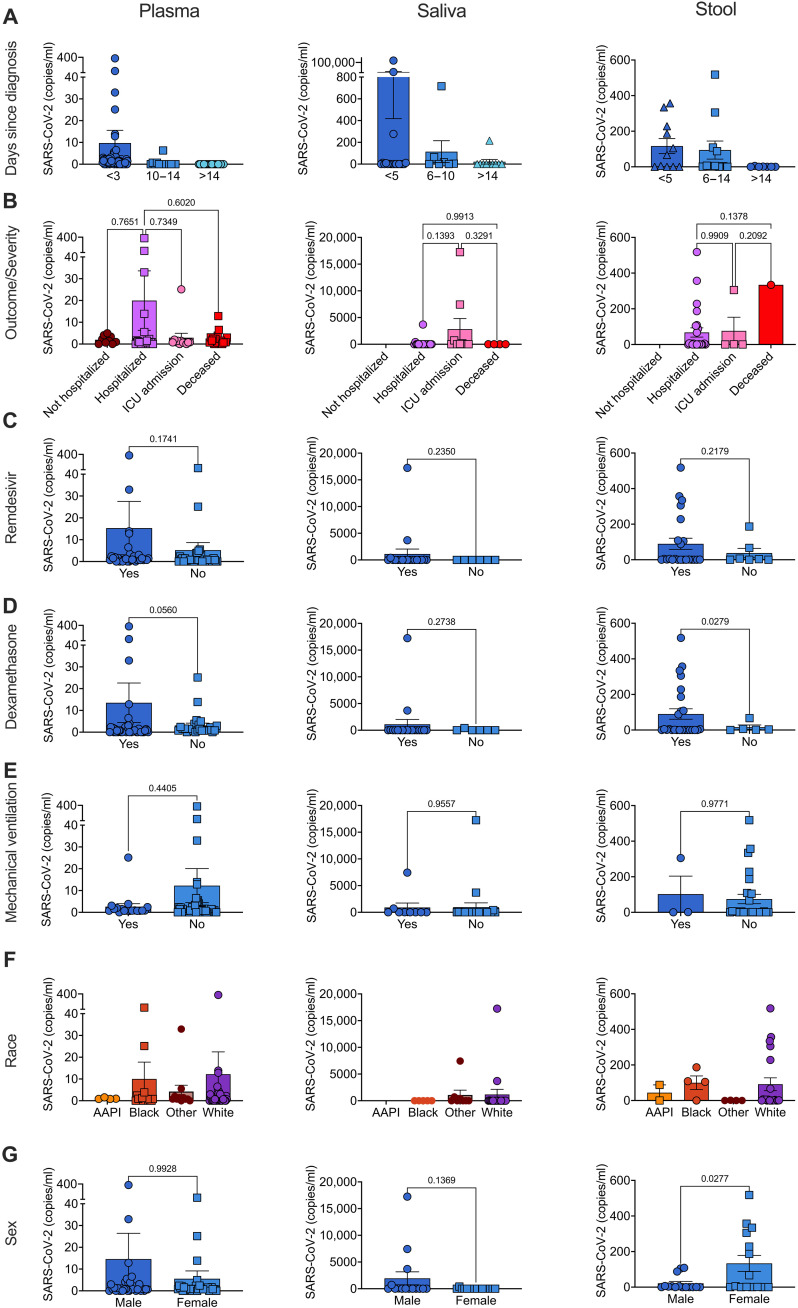
Detection of SARS-CoV-2 in patients with COVID-19 using the ^virus^HB-Chip. Absolute copies per milliliter of SARS-CoV-2 detected in plasma of patients with COVID-19 (left column, *N* = 103), saliva (middle column, *N* = 36), and stool (right column, *N* = 29) showing differences in levels based on (**A**) days since diagnosis, (**B**) outcome/severity, (**C**) remdesivir treatment, (**D**) dexamethasone treatment, (**E**) mechanical ventilation, (**F**) race, or (**G**) sex. AAPI, Asian American Pacific Islander.

We used the Mass General Brigham Biobank to select patients (*n* = 69) that were within 2 to 3 days of a positive SARS-CoV-2 test via nasopharyngeal swab. We found an average of 9.7 copies per milliliter of SARS-CoV-2 and a positivity rate of 72% of samples. We tested two additional cohorts of patients collected at Mass General Hospital further from time of diagnosis. When we tested patient plasma samples (*n* = 14) collected ~8 days from diagnosis, the average dropped to 0.55 copies per milliliter with a positivity rate of 21% of samples tested. Samples collected 20 days from diagnosis (*n* = 20) showed no detectable SARS-CoV-2 in the plasma (Table 1 and Fig. 5A). This suggests that the highest rates of SARS-CoV-2 are seen within the first week after diagnosis. When we considered a threshold of SARS-CoV-2 positivity of more than two ddPCR droplets (as outlined in the Bio-Rad Emergency Use Authorization), we found that 38% of samples collected at ~2.6 days after diagnosis and 7% of samples collected at 10 days after diagnosis were positive for SARS-CoV-2. We saw no detection in plasma samples from SARS-CoV-2–negative patients (Table 1).

**Table 1.** SARS-CoV-2 detection and sample information. For each cohort of patients, the following information is provided from left to right. The type of sample tested, as well as the number of samples in the cohort. The mean times between a positive SARS-CoV-2 PCR test and sample collection for the cohort with the 95% confidence interval are shown in parentheses. The mean sample volumes of the cohort with the 95% confidence interval are shown in parentheses. The mean copies per milliliter of SARS-CoV-2 viral particles determined by ddPCR for the cohort with the 95% confidence interval are shown in parentheses. % Positive is displayed for all samples that contained at least one positive droplet for SARS-CoV-2 by ddPCR and for samples with at least two droplets positive for SARS-CoV-2 by ddPCR (greater than two SARS-CoV-2–positive droplets is the threshold for positivity outlined in Bio-Rad’s Emergency Use Authorization). n/a, not applicable.

Detection of SARS-CoV-2 in COVID-19 patients using the ^virus^HB-Chip					
				% Positive	
**COVID^+^ samples**	**Time from diagnosis (days)**	**Volume tested (ml)**	**Copies per milliliter**	**>1 droplet**	**>2 droplets**
Plasma (*n* = 69)	2.6 (± 0.44)	1.0 (± 0.00)	9.74 (± 11.4)	72%	38%
Plasma (*n* = 14)	7.9 (± 2.28)	0.26 (± 0.04)	0.55 (± 0.88)	21%	7%
Plasma (*n* = 20)	20 (± 2.4)	0.22 (± 0.006)	0.0 (± 0.0)	0%	0%
Saliva (*n* = 36)	8.9 (± 3.3)	0.22 (± 0.04)	3530 (± 6441)	39%	39%
Stool (*n* = 29)	8.4 (± 2.6)	0.67 (± 0.07)	80.2 (± 50.3)	62%	58%
**Healthy samples**	**Time from diagnosis (days)**	**Volume tested (ml)**	**Copies per milliliter**	**>1 droplet**	**>2 droplets**
Plasma (*n* = 5)	n/a	0.25 (± 0.00)	0.0 (± 0.0)	0%	0%
Saliva (*n* = 6)	n/a	0.28 (± 0.00)	0.0 (± 0.0)	0%	0%
Stool (*n* = 4)	n/a	0.20 (± 0.00)	0.0 (± 0.0)	0%	0%

#### Detection rate of intact SARS-CoV-2 in patient saliva and stool samples

Next, we tested whether our platform would also work for viral isolation from other complex biofluids. To briefly highlight our optimization steps, we obtained the highest rate of SARS-CoV-2 capture with our platform when raw stool was diluted in Ficoll-PAQUE (fig. S8A), and particulates were removed through centrifugation (fig. S8B). For saliva samples, we found that undiluted samples processed through the chip gave the highest rates of detection. We additionally tested different saliva collection devices and their impact on SARS-CoV-2 detection in spiked control samples. These devices are known to filter samples and help remove mucosal strands or other potentially confounding particulates. We found that the Salivette showed improved capture efficiency to no device; however, the Pure-SAL showed the highest rates of detection. The Super-SAL device showed little to no detection (fig. S9). This is likely because of the preservative used after collection with this device to stabilize RNA disrupted viral particles. While it was determined that the Pure-SAL collection device resulted in the highest sensitivity for the ^virus^HB-Chip, it should be noted that all saliva samples collected from patients with SARS-CoV-2 at Mass General Hospital used the Salivette device due to established IRB protocols.

To determine the presence of SARS-CoV-2 viral particles in patient samples, we obtained saliva samples from patients confirmed as positive for SARS-CoV-2 (*n* = 36) as well as from healthy controls (*n* = 6). We first verified that in our set of known SARS-CoV-2 negative samples, we had no false positives when we processed saliva or stool through the ^virus^HB-Chip (Table 1). For our samples from patients with SARS-CoV-2, collected within ~10 days of diagnosis, we found an average of 3530 copies per milliliter and a positivity rate of 39% of samples tested. We found no difference when using a cutoff of more than two droplets to determine positivity (Table 1). We additionally observed that the amount of SARS-CoV-2 was highest in the samples we tested that were collected within 5 days of diagnosis, dropping off at 6 to 10 days (Fig. 5A). We tested stool samples from SARS-CoV-2–positive patients (*n* = 29), obtained from ~8 days after diagnosis, and found an average of 80 copies per milliliter of SARS-CoV-2 (Table 1 and Fig. 5A) and persisted for up to 14 days (*n* = 22) (Fig. 5A). Only after 14 days did we see a drop in SARS-CoV-2 in the stool of patients with COVID-19 (*n* = 7). Use of a microfluidic assay like our ^virus^HB-Chip could identify the potential infectivity of different biofluids by determining how many intact viral particles are present in a sample.

### SARS-CoV-2 levels compared to clinical metrics

#### *Patient plasma samples* (*collected within 72 hours*)

To determine whether the viral levels detected using our assay were indicative of later clinical outcomes, we compared detected levels of SARS-CoV-2 in plasma collected within 72 hours to clinical metrics associated with COVID-19, patient demographics, and known comorbidities. We examined the set of 69 plasma samples collected within 72 hours of a positive SARS-CoV-2 PCR test, both because we wanted to determine whether levels measured early could predict later patient outcome and because this set of patients from the Mass General Brigham Biobank had the largest collection of corresponding clinical data. When we examined the severity of disease as defined as 1 (not-hospitalized), 2 (hospitalized), 3 [intensive care unit (ICU) level care], or 4 (deceased), we found that the highest levels of SARS-CoV-2 were seen in severity 2 patients (hospitalized). Unexpectedly, patients that later succumbed to COVID-19 had lower, although not significant, levels of SARS-CoV-2 in their plasma 3 days after diagnosis (Fig. 5B). Patients receiving ACE inhibitors had lower, although not significant, levels of SARS-CoV-2 in the plasma (fig. S10A). Patients that received anticoagulants, remdesivir, or dexamethasone treatment had higher, but not significant, levels of SARS-CoV-2 in the plasma (Fig. 5, C and D, and fig. S10B). Patients that later received supplemental oxygen showed higher levels of plasma SARS-CoV-2, whereas patients requiring mechanical ventilation showed lower levels (Fig. 5E and fig. S10C).

When we examined the demographics of our study population, we found no difference in SARS-CoV-2 levels in plasma depending on the race, ethnicity, or sex of the patient (Fig. 5, F and G). When we examined the age of the patient, we found that we tended to see higher, although not significant, viral levels in patients older than 56 and 65 years old (fig. S11). In addition, viral levels among groups of patients with comorbidities putting them at risk for more severe outcome (patients with type 2 diabetes, hypertension, obesity, or asthma) showed no statistical differences in SARS-CoV-2 plasma levels (fig. S12, A to E). Patients with immunodeficiency also showed higher, but not significant, levels of SARS-CoV-2 in the plasma (fig. S12C). This suggests that viral levels in plasma may be indicative of patients in need of interventional therapies for COVID-19 independent of traditional comorbidities that put patients at risk for developing severe disease. We found an increased, but not significant, levels of SARS-CoV-2 in the plasma of patients that reported symptoms of fever, shortness of breath, or fever with shortness of breath (fig. S13, A to C). Patients with more severe symptoms, including pneumonia or cytokine release syndrome, had higher (but not significant) levels of SARS-CoV-2 in the plasma (fig. S13, D and E). Patients with acute kidney failure showed no difference in SARS-CoV-2 levels (fig. S13F), and those with other viral pneumonias had lower (but not significant) levels of SARS-CoV-2 in the plasma (fig. S13G).

#### Patient saliva and stool samples

We also tested whether SARS-CoV-2 levels in patient saliva or stool correlated with known clinical metrics available (Fig. 5). The available data for this patient set included demographics of sex and race but not age. Patients with a severity score of 3 (requiring ICU level care) had the highest levels of SARS-CoV-2 in their saliva (Fig. 5B). In addition, patients presenting to the emergency room with pneumonia or respiratory distress had the highest levels of SARS-CoV-2 in the saliva (fig. S14). We saw no difference in levels of patients that were later admitted to the ICU or required mechanical ventilation in saliva SARS-CoV-2 levels (Fig. 5B). There were higher, but not significant, levels of SARS-CoV-2 in saliva of patients that later were treated with remdesivir or dexamethasone (Fig. 5, C and D), which was also observed in patient plasma. In saliva samples, we saw no difference between samples collected on the basis of sex or race (Fig. 5, F and G).

We saw no difference in saliva SARS-CoV-2 levels of patients that were later admitted to the ICU or required mechanical ventilation (Fig. 5, B and E). There were higher, but not significant, levels of SARS-CoV-2 in stool of patients that later were treated with remdesivir or dexamethasone (Fig. 5, C and D), which was also observed in patient plasma and saliva. This indicates that viral levels in patient samples during acute infection could be predictive of later patient therapeutic needs. In stool samples, we saw a significantly higher viral levels in female patients but no difference between samples collected on the basis of patient race (Fig. 5, F and G). Within stool samples tested, all male samples were from severity 2 (hospitalized) patients, while within female patients, 10 were from severity 2, 3 from severity 3 (ICU level care), and 1 from severity 4 (deceased). Notably, two of the three stool samples with >300 copies per milliliter were from severity 3 and 4 patients, indicating that the higher levels we observed in female stool samples was likely due to the higher severity of the female patients recruited.

#### Serial monitoring in patient plasma

**Fig. 6.**
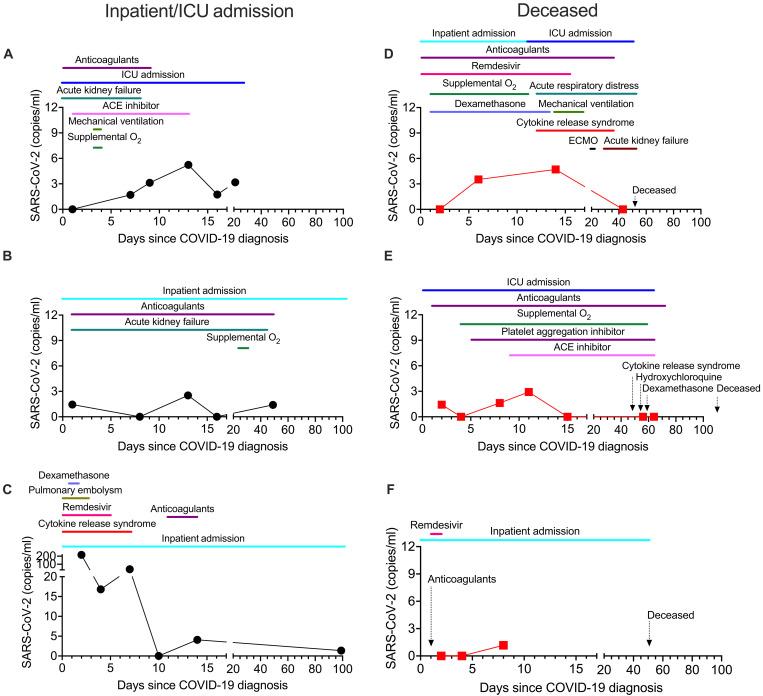
Serial monitoring of SARS-CoV-2 in plasma. (**A** to **F**) SARS-CoV-2 copies per milliliter were detected using the ^virus^HB-Chip in plasma samples collected over the course of treatment. Patients that survived are designated by black circles (A to C, left) and patients that are deceased by red boxes (D to F, right). Conditions and treatments are designated by date using colored lines or by arrows for treatments or conditions lasting only 1 day. ECMO, extracorporeal membrane oxygenation; copies/ml, copies per milliliter.

To better understand how our assay could be used to study viral infection over time, we sought to examine patient samples collected serially during disease. We obtained serial plasma samples collected through the Mass General Brigham Biorepository for 10 patients with SARS-CoV-2 with a variety of outcomes. Three patients that were category 2 severity (hospitalized) or 3 severity (requiring ICU level care) displayed maintained or increasing levels SARS-CoV-2 of SARS-CoV-2 in plasma despite therapeutic interventions (Fig. 6, A to C). One patient with very high levels of SARS-CoV-2 in plasma initially (>200 copies per milliliter) reported to the emergency room with a pulmonary embolism and cytokine release syndrome (Fig. 6C). Three patients that were category 4 severity (died following SARS-CoV-2 infection) also maintained levels of SARS-CoV-2 in plasma despite therapeutic interventions past 10 to 14 days and fell to zero before their death (Fig. 6, D to F). This demonstrates that our assay can track viral levels in plasma across multiple time points in patients.

## DISCUSSION

### General summary

Our results show that we can reliably use our ^virus^HB-Chip for capture of viral particles from a variety of patient biofluids. We have found intact viral particles in plasma, saliva, and stool of patients with SARS-CoV-2, with the highest levels present in saliva samples. Higher levels in saliva may arise from the proximity of saliva samples to viral replication in the nasopharyngeal tissue (*11*, *12*, *14*, *19*). Plasma is a particularly challenging biofluids to isolate extracellular vesicles or viruses using immunoaffinity capture (*33*–*36*). Despite these challenges, we demonstrated that our microfluidic chip can reliably capture as few as 3 viral particles per milliliter of plasma. Our assay can measure intact viral particles in plasma, saliva, and stool of patients with COVID-19. This suggests our assay is particularly well suited for detection of rare viral particles, even in biofluids that are traditionally thought difficult for capture of particles or RNA free of confounding factors.

### Limitations

To determine the sensitive and specificity of our assay, we relied on collection of samples from patients that had a positive PCR test from a nasopharyngeal swab. However, given that we do not fully understand which biofluids will contain SARS-CoV-2, as well as how low these levels may be, it is challenging to understand which samples may be bellow detection versus having no virus. However, by examining our limit of detection in spiked samples, including in blinded samples, we can be confident that we are detecting as low as 3 copies per milliliter. In ~40% of our plasma samples collected 2 to 3days following a positive SARS-CoV-2 PCR, we detected as few as 1 viral copy per milliliter of plasma. This is just below our limit of detection performed with spiked samples but not unexpected given how low we can reliably detect virus and the sensitivity of our ddPCR readout. However, the threshold for positivity defined by the Bio-Rad assay is two positive droplets. Because of this, we included these samples in our analysis but have also noted the percentages of patients where we are seeing at least two positive droplets. When comparing viral levels to later outcome in saliva and stool, we did not have any nonhospitalized patients because of our collection of samples through clinical studies. While we do see a trend of higher levels in severity 2 (hospitalized) and 3 (requiring ICU care) in the saliva and stool of patients, we do not have severity 1 (nonhospitalized) samples with which to compare for these sample types. The presence of anti–SARS-CoV-2 spike protein antibodies or shed ACE2 in the plasma could limit the ability of our assay to detect intact viral particles. These antibodies or shed ACE2 proteins could act as competitive binders to the ACE2 our ^virus^HB-Chip uses to capture intact SARS-CoV-2, limiting our ability to detect intact virus in their presence.

### Capture of different variants

Our device has demonstrated efficient capture of all variants tested. We found that variants that results in higher binding affinities to ACE2 (Delta variant) are recovered more efficiently by our device (*79*, *82*). This suggests that we could use our device to screen for previously unidentified variants in individual or pooled samples to screen for unknown variants that bind to ACE2 more tightly. One could potentially use recombinant ACE2 or spike protein at increasing concentrations to wash out pools of variants that bound at different avidities to ACE2. These samples could then be sequenced to reveal whether there were any unknown or new mutations or variants associated with higher binding. This would allow for tracking of variants across communities and patients because of the ability of our assay to capture intact viral particles. If viral variants mutated to a degree wherein capture via our ACE2^ENG^ receptor was not sufficient, our capture moiety can be adjusted using the universal biotin/neutravidin linker on our microfluidic device.

### Clinical implications of our test

Most clinical testing has relied on qPCR testing of nasopharyngeal swabs with a push for testing of saliva as an alternative. There have been some conflicting reports of how much virus can be found in the plasma of patients with COVID-19. Some studies have found none, others between 10 and 15% of samples (*7*). Here, we found intact virus present in the plasma of 38% of our patient samples tested, demonstrating a clear improvement of viral capture in plasma in addition to our ability to distinguish free viral RNA from intact viral particles. Some studies have suggested that patients with a more severe prognosis have higher levels of virus in plasma compared to those with better outcome, even to later time points (*98*). In the patient samples we tested within 3 days of a positive nasopharyngeal swab PCR test, we found that patients who later succumbed to COVID-19 had lower levels of virus present in the blood. This result when considered with our ability to measure intact virus in saliva and stool further underscores the clinical need to detection of intact viral particles, to differentiate from shed viral RNA, when examining whether viral RNA can be a predictive marker of patient outcome. Our assay was able to detect SARS-CoV-2 below 12 copies per milliliter across multiple variants, suggesting that its ability to detect SARS-CoV-2 is agnostic of the variant present in the clinical sample. We also found that the levels of SARS-CoV-2 in the plasma were not associated with clinical comorbidities that are commonly associated with an increased risk of severe COVID-19 (*87*, *88*). This suggests that the ability to detect intact virus is independent of these other clinical metrics. In addition, because the detection of intact viral particles is independent of other predictors of Long Covid, our assay could be used in the future to understand how the viral kinetics of SARS-CoV-2 can lead to Long Covid in some individuals with COVID-19.

Our assay also detected intact SARS-CoV-2 in 39% of patient saliva samples tested and 59% of patient stool samples tested. No other groups have monitored viral levels in stool of patients with COVID-19. Levels of SARS-CoV-2 were higher in the saliva and stool of patients with worse outcomes, suggesting that they may be more indicative of outcome. Measurements of intact viral particles in saliva, circulating in plasma, and presence in stool also suggest the viral kinetics of SARS-CoV-2 spread throughout the body. Saliva and stool also displayed SARS-CoV-2 positivity at time points later than in plasma samples, suggesting that after initial infection and spread, viral infection is present in these tissues for longer periods. This could explain the development of end organ damage and/or Long Covid in some infected individuals. Our viral detection in plasma, saliva, or stool could be used in conjunction with other clinical indicators, to determine patients that would benefit from COVID-19 therapies and interventions before disease progression. In addition, by testing the presence of intact viral particles in different biofluids or organs, we can further understand the role of SARS-CoV-2 viral kinetics and Long Covid.

### Implications for use for other viruses

Because of the ability of our assay to detect intact viral particles at ultralow levels in plasma, there are implications for the adaptation of our technology for other viral diseases. HIV research and clinical therapy rely on the ability to monitor viral load in blood during treatment. Detection of virus in plasma in coordination with CD4^+^ T lymphocyte count has been an essential tool for monitoring patients with HIV to determine therapeutic efficacy as well as potential transmission of the virus. Patients with an HIV viral load <20 viral copies per milliliter measured by PCR are considered undetectable (*99*) and a viral load <80 copies per milliliter are considered untransmittable (unable to transmit the virus to partners) (*100*). By using a similar method as we have for capturing SARS-CoV-2 from plasma with the ^virus^HB-Chip using its receptor for viral entry, researchers could isolate intact HIV from patient plasma or other sources. Current clinical methods of viral detection in plasma are sufficient to determine whether someone on antiretroviral therapy has an undetectable viral load or can be considered untransmittable. These assays range from a limit of detection from 20 to 200 copies per milliliter (*99*). However, initiatives into curing HIV through vaccines, stem cell transplants, or other methods would benefit from the ability to detect residual virus in the blood at even lower levels. This would greatly increase the ability for researchers to better measure whether their investigational therapies or cures are working to eliminate the virus. Clinical therapy for HIV could also benefit by showing that in patients on antiretroviral therapy, viral levels are not detectible in an assay with a limit of detection between 10 and 100 times lower than current clinical assays. In addition, if a therapy may become less effective, researchers or clinicians could detect a rise in plasma HIV levels much sooner. This would allow them to better track and understand how and when therapies are effectively reducing viral levels in the blood. Our assay could additionally be quickly translated for other viral pandemics in the future, using the ability of our assay to capture intact viral particles used to understand which patients have active viral infections versus those shedding viral RNA during recovery.

## MATERIALS AND METHODS

### Microfluidic device

For this study, we used a multichannel, single-inlet and single-outlet, microfluidic device that we refer to as the “herringbone chip” (HB-Chip) (*91*, *94*, *95*, *101*, *102*). We selected this device because of its rapid scalability and ability to isolate rare particles of interest. Injection molded HB-Chips were manufactured by thinXXS Microtechnology (Germany).

### Microfluidic device functionalization 

Plastic HB-Chips were inspected for debris and imperfections using microscope (Evos XL Core, AMEX1000). A 20 mM *p*-phenylenediamine (Sigma-Aldrich, P6001) solution in 1 M hydrochloric acid (HCl, Sigma-Aldrich, H1758) and a 20 mM sodium nitrite (Sigma-Aldrich, 237213) solution were reacted with EZ-Link NHS-Biotin (final concentration of 10 mM, Thermo Fisher Scientific, 20217) for 30 min at room temperature to form a biotin aryl-diazonium salt. Devices were then flushed with 200 μl of the biotin aryl-diazonium solution through the inlet and exposed to UV light using a UV light bed (UVP Transilluminator PLUS, 95042001) set to “High” for 10 min. UV light allows for the creation of biotin aryl radical intermediates that then react with the plastic surface of the device. Devices were then flushed with 500 μl of ethanol (ethyl alcohol, Sigma-Aldrich, 493546) to remove bubbles, followed by 500 μl of PBS (Gibco, 10010049) through the inlet. Another 200 μl of the biotin aryl diazonium intermediate solution was flushed through the outlet of each device followed by another 10-min UV exposure. Devices were flushed with 500 μl of ethanol to remove bubbles, followed by 500 μl of PBS for immediate use or 10 ml of air to dry them. Dried herringbone chips were then stored at 25°C in a vacuum desiccator until used. To rehydrate, dried herringbone chips were flushed with 500 μl of ethanol and 500 μl of PBS. Devices were then flushed with 200 μl of a 0.01667% solution of streptavidin nanoparticles (Spherotech, SVP01-008-5) in PBS through the inlet of the devices. After a 15-min incubation, another 200 μl of streptavidin nanoparticles were flown through the outlet of the device. Chips were then used immediately for viral capture (*90*) 

### Protein biotinylation for viral capture

ACE2^ENG^ (*92*), Wild Type ACE2 (BioLegend, 792008), nonspecific IgG (BioLegend, 401402), or an anti-spike protein targeting antibody (Thermo Fisher Scientific, PA5-114528) were incubated at room temperature while rotating with Biotin PEG SCM 2 kDa (Creative PEGworks, PJK-1900) for 2 hours at a molar ratio of biotin linker:antibody of 20:1. Excess biotin linker was removed using Zeba Desalting Columns (Thermo Fisher Scientific, 89882). Capture proteins were then aliquoted for single use and stored at −80°C.

### Spike protein expression construct

The codon-optimized spike gene was PCR amplified from a plasmid obtained from Sino Biological (VG40589- UT). This gene encodes a version of the spike protein with amino acid identical to QHD43416.1 GenBank entry. The resulting gene, S-D614-ΔC-HA, lacks the C-terminal 19 amino acids in the spike protein and contains a C-terminal hemagglutinin (HA) tag (YPYDVPDYA). This construct was cloned in a PiggyBac (PB) vector (System Bio) to make the PB-S-D614-ΔC-HA vector. The PB-S-D614-ΔC-HA vector was then subjected to Quickchange XL Site-directed Mutagenesis (Agilent) with the primers 5′CTCAGTACAGTTCACACCCTGGTAGAGCACAGC3′ and 5′GCTGTGCTCTACCAGGGTGTGAACTGTACTGAG3′ to obtain the PB-S-G614-ΔC-HA vector. The resulting D614G mutation was confirmed by Sanger sequencing.

### Pseudotyped virus production and quantification

human embryonic kidney–293T cells were seeded in T150 or T75 flasks at ~50% confluency the night before transfection. The next day, the cells were cotransfected with pSin-DsRed-IRES-Puro, the psPAX2 packaging vector, and one of the spike-expressing PB plasmids depending on the experiment performed. A 1:1:1 molar ratio of all three plasmids for a total DNA concentration of 20 or 40 μg (T75 versus T150 flask) was used to transfect the cells using the TransIT-LT1 Transfection Reagent (Mirus Bio). Three days later, the supernatant was collected and spun at 350*g* for 10 min at 4°C and then filtered through a 0.45-μm filter. The virus was then pelleted by ultracentrifugation at 100,000*g* over a 20% sucrose cushion. The virus was resuspended in sterile PBS and then quantified using the Lenti-XTM p24 Rapid Titer Kit (Takara Bio). Aliquots were frozen at −80°C for future use.

### Viral propagation

All work with infectious SARS-CoV-2 was conducted in Biosafety Level-3 (BSL3) conditions at the University of California San Diego (UCSD) following the guidelines approved by the Institutional Biosafety Committee. SARS-CoV-2 viruses were obtained from BEI (WA1: BEI no. NR-52281, B.1.351 (Beta variant): BEI no. NR-54009, B.1.617.2 (Delta variant): BEI no. NR-55611) or isolated at UCSD from consented patient samples (B.1.1.7 (Alpha variant) under UCSD IRB no. 200477 and Omicron BA.1 and BA.5.1 under UCSD IRB no. 160524). Isolates have been described previously: B.1.1.7 (Alpha variant) (*103*), (original sequence GISAID no. EPI_ISL_751801), Omicron BA.1 (*103*) (original sequence GISAID no. EPI_ISL_8186377), and Omicron BA.5.1 (*104*) (original sequence GISAID no. EPI_ISL_14243842). Viral stocks were propagated on TMPRSS2-VeroE6 cells (Sekisui XenoTech) in Dulbecco’s modified Eagle’s medium (DMEM) with l-glutamine + 2 to 3% fetal bovine serum (FBS) + penicillin/streptomycin (pen/strep) + 10 mM Hepes. Supernatants were harvested and centrifuged at 1000*g* 10 min to remove cellular debris and stored at −80°C. SARS-CoV-2 stocks were titered by fluorescent focus assay using antinucleocapsid primary antibody (GeneTex, gtx135357) and by median tissue culture infectious dose (TCID_50_) on TMPRSS2-VeroE6 cells and confirmed by whole-genome sequencing.

Human respiratory syncytial virus subgroup A/strain A2 [American Type Culture Collection (ATCC) VR-1540] was propagated on HeLa cells in DMEM + 2% FBS + 1% pen/strep. Human respiratory syncytial virus subgroup B/strain 18537 (ATCC VR-1580) was propagated on HEp-2 cells in minimum essential medium (MEM) + 2% FBS + 1% GlutaMax +1% pen/strep. Cell pellets were harvested, resuspended in DMEM, and subject to three freeze-thaw cycles. Infectious virus was quantified by TCID_50_ assay on HeLa and HEp-2 cells, respectively.

Influenza viruses A (H3N2, ATCC VR-1938) and B (Victoria, ATCC VR-1784) were propagated on Madin-Darby canine kidney (MDCK) cells (ATCC CCL-34) in DMEM + 0.5% bovine serum albumin + 1% pen/strep + TPCK-trypsin (2 μg/ml) and titered by plaque assay on MDCK cells. Human coronavirus OC43 (ATCC VR-1558) was propagated on HCT-8 cells (ATCC CCL-244) in RPMI 1640 + 2% horse serum + 1% pen/strep. Supernatants and cell pellets were saved, and pellets were freeze/thawed for three rounds, remixed with supernatant, clarified by centrifugation at 400*g* for 10 min at 4°C. Stocks were titered by TCID_50_ assay on HCT-8 cells with staining for nucleocapsid protein (antinucleocapsid, Sigma-Aldrich, MAB9013). Human coronavirus 229E (ATCC VR-740) was propagated on MRC-5 cells (ATCC CCL-171) in MEM + 10% FBS + 1%pen/strep + 10 mM Hepes and titered by plaque assay on MRC-5 cells.

### Viral inactivation

Viral stocks were heat inactivated in a heat block at 65°C for 30 min. UV inactivation was performed in a UV cross-linker (Analytik Jena). A thin layer of supernatant (3 to 4 ml) was spread evenly in a 10-cm petri dish, and 400 mJ/cm^2^ of UV_254_ was applied. Virus inactivation was confirmed by attempting to culture at least 10% of the inactivated product on their respective permissive cell lines. Inactivated viruses were aliquoted and stored at −80°C and shipped on dry ice.

### Nanoparticle tracking analysis

Multi-laser NTA was performed using the ViewSizer 3000 (HORIBA) on heat and UV-C inactivated SARS-CoV-2. Inactivated virus was diluted 200× with PBS. Sample cuvettes were washed two times with 200 μl of diluted viral samples. Diluted samples (400 μl) were loaded into the cuvette, and measurements were taken as follows. Twenty-five 10-s videos were recorded of each sample with 5 s of sample mixing at 1400 rpm between measurements. Cuvettes were washed five times with PBS between samples and with dilute detergent, followed by Milli-Q water and 100% ethanol between uses. Analysis was then performed using the ViewSizer3000 software using the Exosome MPTA method following standard settings to determine size and concentration of viral particles.

### TEM imaging

UV- or heat-inactivated SARS-CoV-2 samples were fixed with 4% glutaraldehyde and diluted 1:10 with 1× PBS. Each sample (10 μl) was transferred onto a 200-mesh Formvar/carbon-coated nickel grid and allowed to adsorb for 15 min. Grids were blotted (to remove excess suspension solution) and contrast stained for 30 to 60 s using 2% uranyl acetate solution. Grids were blotted again, rinsed once with filtered distilled deionized water, and, after blotting residual liquid, allowed to air dry before analysis. Examination of preparations was done using a JEOL JEM 1011 transmission electron microscope at 80 kV. Images were collected using an AMT digital imaging system with proprietary image capture software (Advanced Microscopy Techniques, Danvers, MA).

### Direct stochastic optical reconstruction microscopy

SARS-CoV-2 (Delta variant, UV-C inactivated) were captured on the surface of ONI’s EV-profiler Application Kit (EV-MAN-1.0) using ACE2^ENG^. S3 reagent was added to the chip for 10 min to coat the surface, and chip was then washed with W1. ACE2^ENG^ (25 μg/ml) were added to the chip lane and incubated for 10 min. After washing, the chip was blocked for 10 min using N1 reagent. Approximately 6 × 10^7^ viral particles were added to the chip and incubated for 50 min at room temperature. Unbound viral particles were washed and chip was fixed with F1 solution. After washing, samples were incubated for 50 min with an anti-spike protein antibody (Thermo Fisher Scientific, PA5-114528) labeled with CF-568 (MilliporeSigma, MX568S100-1KT). Samples were washed, fixed with F1 solution, and washed again. For dSTORM buffer preparation, 5 μl of B^3^B buffer was mixed with B^3^A buffer, and 40 μl was added in the lane. Imaging was immediately performed in the ONI Nanoimager using a TIRF illumination angle and images were analyzed using the CODI software. S3, W1, N1, F1, B^3^B and B^3^A solutions were all provided in the kit.

### Pseudovirus and inactivated SARS-CoV-2 processing

All experiments were conducted under a Mass General Brigham–approved Institutional Biosafety Protocol. Inactivated SARS-CoV-2 is described above. For pseudovirus or inactivated SARS-CoV-2 capture, virus was spiked into healthy donor plasma (Innovative Research, IPLASK2EDTAUNIT), saliva (Innovative Research, IR100044P-50ML), or stool (collected from healthy individuals under a Massachusetts General Brigham secondary use protocol no. 2023P000215). All samples were stored at −80°C and thawed at 37°C in a water bath or heat block. For whole-blood experiments, blood was donated from healthy donors under IRB no. 2009-P-000295 and used within 1 hour for experiments. Virus was diluted in healthy donor plasma or blood according to the notes in each figure legend.

For all devices, 200 μl of a solution of capture protein/antibody (25 μg/ml) was added to inlet of each device and incubated at room temperature for 30 min. Then, 200 μl of the same solution was flown through the outlet of the same device. After a 4-hour incubation at room temperature or overnight incubation at 4°C, devices are blocked with 200 μl of Intercept (TBS) Blocking Buffer (Licor, 927-60001).

The diluted virus was passed through the herringbone chip at 1 ml/hour using a 1-ml syringe (Air-Tite, ML1). Devices were then washed with 1.5 ml of PBS flown through at 1.5 ml/hour. For experiments with in-solution antibodies, IgG or anti-spike protein antibodies or ACE2^WT^ protein were added to healthy donor plasma spiked with virus. After a 1-hour incubation while rotating, the plasma-virus-antibody solution was run through blocked herringbone chips without antibodies.

For experiments with RNase A treatment, 0.5 ml of RNase A (6.25 μg/ml; Thermo Fisher Scientific, EN0531) was flown through the device after the sample at 1.5 ml/hour. RNase A was incubated in the chip for 30 min at 37°C. The chips were then rinsed with 1.5 ml of PBS at 1.5 ml/hour containing RNase inhibitor (0.8 U/μl; Takara, 2313A).

### Clinical sample processing

All experiments were conducted under a Mass General Brigham–approved Institutional Biosafety Protocols (no. 2020P000804, no. 2007P002451, and no. 2023P000215). For all ^virus^HB-Chips, ACE2^ENG^ protein and Intercept (TBS) Blocking Buffer were added to each device as mentioned above. Clinical plasma, saliva, or stool patient samples were stored in −80°C upon receiving and were thawed in a 37°C water bath or heat block with beads. Plasma, stool, or saliva samples were collected under a secondary use protocol approved by the Massachusetts General Brigham IRB protocol no. 2023P000215. For stool samples, 2-ml Ficoll-Paque Premium (GE Healthcare, 17-5442-02) was added per gram of stool and then vortexed for 1 min at medium speed. Stool was then centrifuged at 1000*g* for 10 min to remove solid debris and the supernatant was collected for processing. Samples were processed in BSL2 biosafety cabinets following BSL3 standards. Samples were loaded by pipette into 1-ml syringes. Samples were flown through device at 1 ml/hour. Devices were then washed with 1.5 ml of PBS using 3-ml syringes (BD, 309657). Devices were initially washed with 600 μl of PBS flown through at 1 ml/hour and then with 900 μl of PBS flown through at 1.5 ml/hour. This adjustment allowed remaining sample to be flushed out of devices at the same flow rate.

### RNA extraction

For all patient samples, RNA was extracted from devices using the MagMAX Viral/Pathogen Nucleic Acid Isolation Kit (Applied Biosystems, A42352). For each device, a solution of 530 μl of Binding Solution (from A42352) + 20 μl of Total Nucleic Acid Magnetic Beads (from A42352) + 10 μl of Proteinase K (from A42352) was flown through 16 times by pushing between syringes attached to the inlet and outlet port of devices using syringe pumps. RNA was then isolated per the manufacturer’s manual protocol (Applied Biosystems, A42352) or automated protocol using the Kingfisher Flex System (Thermo Fisher Scientific, 5400630). For some pseudovirus and inactivated virus samples, RNA was extracted from devices using the Zymo Quick-RNA Microprep Kit (Genessee Scientific, R1050). For each device, 400 μl of TRI reagent (Genessee Scientific, R2050-1-200) was flown through 15 times by manually pushing between syringes attached to the inlet and outlet ports of devices. RNA was then isolated per the manufacturer’s protocol.

### One-step reverse transcription and ddPCR

RNA copy numbers were measured using the One-Step RT-ddPCR Advanced Kit for Probes (Bio-Rad, 1864021). All SARS-CoV-2 RNA levels were measured using the 2019-nCoV CDC ddPCR Triplex Probe Assay (Bio-Rad, 10000064743) and non–SARS-CoV-2 viruses were measured using integrated DNA technology (IDT) primer/probe mixes respective to each virus, noted below. Reactions were performed using 11 μl of RNA per reaction with 1 μM primers (final concentration) and a primer:probe ratio of 4:1. Droplet generation was performed on the QX200 AutoDG, PCR amplification on the C1000 Touch Thermal Cycler, droplet reading on the QX200 Droplet Reader, and analysis using the QX Manager Software v2.0.

### Primer/probe sequences

#### SARS-CoV-2

2019-nCoV CDC ddPCR Triplex Probe Assay (Bio-Rad, 10000064743)

2019-nCoV_N1-F: GACCCCAAAATCAGCGAAAT

2019-nCoV_N1-R: TCTGGTTACTGCCAGTTGAATCTG

2019-nCoV_N1-Probe: FAM-ACCCCGCATTACGTTTGGTGGACC-IABkFQ

2019-nCoV_N2-F: TTACAAACATTGGCCGCAAA

2019-nCoV_N2-R: GCGCGACATTCCGAAGAA

2019-nCoV_N2-Probe-1: FAM-ACAATTTGCCCCCAGCGCTTCAG-IABkFQ

2019-nCoV_N2-Probe-2: HEX-ACAATTTGCCCCCAGCGCTTCAG-IABkFQ

RPP30-F: AGATTTGGACCTGCGAGCG

RPP30-R: GAGCGGCTGTCTCCACAAGT

RPP30-Probe: HEX-TTCTGACCTGAAGGCTCTGCGCG-IABkFQ

#### H3N2 influenza A

IAV H3N2 F: AAGACCAATTCTGTCACCTCTGA.

IAV H3N2 R: CAAAGCGTCTACGCTGCAGTCC.

IAV H3N2 Probe: FAM 5′ TTTGTTTTCACGCTCACCGT-IABkFQ.

#### Influenza B

IBV F: GAGACACAATTGCCTACCTGCTT

IBV R: TTCTTTCCCACCGAACCAAC

IBV Probe: FAM-AGAAGATGGAGAAGGCAAAGCAGAACTAGC-IABkFQ

#### RSV-A

RSV-A F: CTTGATTCCTCGGTGTACCTCTGT

RSV-A R: CTCAATTTCCTCACTTCTCCAGTGT

RSV-A Probe: FAM-TCCCATTATGCCTAGGCCAGCAGCA-IABkFQ

#### RSV-B

RSV-B F: TTGGTTTCTTGGTGTACCTCTATAC

RSV-B R: TTCCTAACTTCTCAAGTGTGGTCCTA

RSV-B Probe: FAM-TCCCATTATGCCTAGACCTGCTGCATTG-IABkFQ

#### CoV-OC43

CoV-OC43 F: ATGTTAGGCCGATAATTGAGGACTAT

CoV-OC43 R: AATGTAAAGATGGCCGCGTATT

CoV-OC43 Probe: FAM-CATACTCTGACGGTCACAAT-IABkFQ

#### CoV-229E

CoV-229E F: TTCCGACGTGCTCGAACTTT

CoV-229E R: CCAACACGGTTGTGACAGTGA

CoV-229E Probe: FAM-TCCTGAGGTCAATGCA-IABkFQ

### Statistical analysis

All graphics were made, and all statistical analysis was done using GraphPad Prism. Unless otherwise noted, bar graphs represent the means ± SEM. For comparing statistical differences between means of multiple samples, a standard two-way analysis of variance (ANOVA) was used. For comparisons between paired groups, a standard two-way ANOVA was used. For all comparisons, *P* values were corrected for multiple comparisons using Tukey’s multiple comparisons test. Correlations were done using a Spearman correlation. Statistical tests used and *P* values are noted in figures and figure legends.
