# Supplementary material for: Ultrasensitive detection of intact SARS-CoV-2 particles in complex biofluids using microfluidic affinity capture
Source: Sci Adv. 2025 Jan 10;11(2):eadh1167. doi: 10.1126/sciadv.adh1167 (PMC11721714; doi:10.1126/sciadv.adh1167)
Supplement: Supplementary file 1 — Supplementary Text Figs. S1 to S15 Tables S1 and S2 Legend for movie S1 [file sciadv.adh1167_sm.pdf]

Science Advances
AAAS

Supplementary Materials for

Ultrasensitive detection of intact SARS-CoV-2 particles in complex biofluids using microfluidic affinity capture

Daniel C. Rabe *et al.*

Corresponding author: Shannon L. Stott, sstott@mgh.harvard.edu;
Genevieve M. Boland, gmboland@mgh.harvard.edu

Sci. Adv. **11**, eadh1167 (2025)
DOI: 10.1126/sciadv.adh1167

The PDF file includes:

Supplementary Text
Figs. S1 to S15
Tables S1 and S2
Legend for movie S1

Other Supplementary Material for this manuscript includes the following:

Movie S1

Supplementary Text

Extended Acknowledgments

The following reagent was deposited by the Centers for Disease Control and Prevention and obtained through BEI Resources, NIAID, NIH: SARS-Related Coronavirus 2, Isolate USA-WA1/2020, NR-52281. The following reagent was obtained from UC San Diego: SARS-Related Coronavirus 2, Isolate hCoV-19/USA/CA_UCSD_5574/2020 (Lineage B.1.1.7, Alpha Variant), contributed by Dr. Alex Clark and Dr. Aaron Carlin and the UC San Diego EXCITE laboratory. Virus was isolated from a nasopharyngeal swab obtained by Dr. Louise Laurent under UCSD IRB #200477. The following reagent was obtained through BEI Resources, NIAID, NIH: SARS-Related Coronavirus 2, Isolate hCoV-19/South Africa/KRISP-K005325/2020, NR-54009, contributed by Alex Sigal and Tulio de Oliveira. The following reagent was obtained through BEI Resources, NIAID, NIH: SARS-Related Coronavirus 2, Isolate hCoV-19/USA/PHC658/2021 (Lineage B.1.617.2; Delta Variant), NR-55611, contributed by Dr. Richard Webby and Dr. Anami Patel. The following reagent was obtained from UC San Diego: SARS-Related Coronavirus 2, Isolate hCoV- 19/USA/CA-SEARCH-59467/2021 (Lineage BA.1; Omicron Variant), contributed by Dr. Alex Clark and Dr. Aaron Carlin and the UC San Diego CALM and EXCITE laboratories. The following reagent was obtained from UC San Diego: SARS-Related Coronavirus 2, Isolate hCoV-19/USA/CA-CFAR-033518/2022 (Lineage BA.5.1, Omicron Variant), contributed by Dr. Alex Clark and Dr. Aaron Carlin with help from the San Diego Center for AIDS Research (SD CFAR). Inactivated viruses were produced with help from the UC San Diego EXCITE and CALM Laboratories, from the San Diego Center for AIDS Research (SD CFAR), and from the RADx-Radical Data Coordination Center (DCC) at University of California San Diego. SD-CFAR is an NIH-funded program (P30 AI036214), supported by the following NIH Institutes and Centers: NIAID, NCI, NHLBI, NIA, NICHD, NIDA, NIDCR, NIDDK, NIGMS, NIMH, NIMHD, FIC, and OAR. NIH RADx-rad DCC is funded under NIH grant# 1U24LM013755-01.

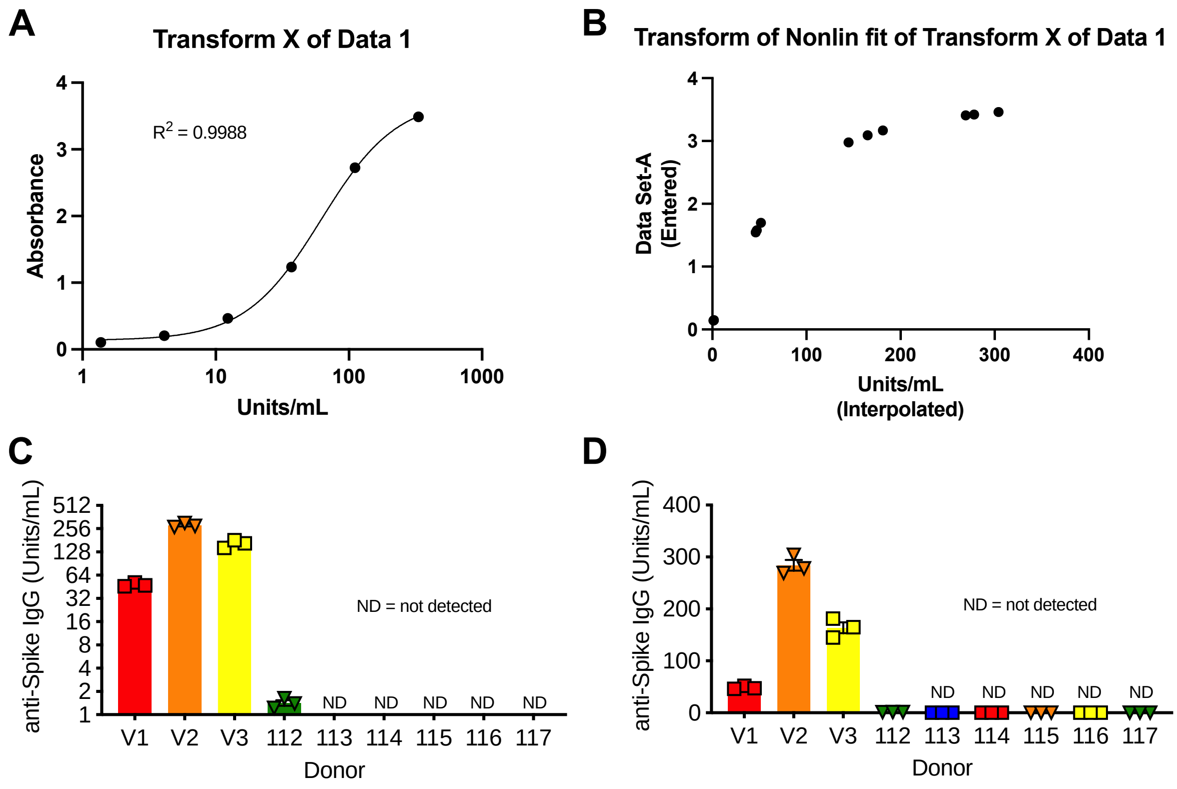

Figure S1: Anti-Spike IgG levels in healthy donor plasma

(A-D) An anti-SARS-CoV-2 Spike protein antibody ELISA was used to determine levels of anti-Spike antibodies in healthy donor plasma. Healthy donor plasma was tested from 6 commercially purchased plasma samples (112 – 117) as well as 3 individuals who had received a SARS-CoV-2 vaccine (V1 – V3) as a positive control. **(A)** A standard curve of anti-Spike antibodies (Units/mL) were measured using an ELISA and $\log_{10}$ transformed known concentrations were graphed on the x-axis against the absorbance on the y-axis and fit to a nonlinear fit curve. **(B)** Interpolated values (x-axis, Units/mL) found using a non-linear curve fit are shown for samples, graphed against their absorbance values (y-axis). **(C-D)** Anti-Spike IgG values for each sample are shown on either a $\log_2$ scale **(C)** or a linear scale **(D)**. ND is used to mark plasma samples in which anti-Spike antibodies were not detected (ND).

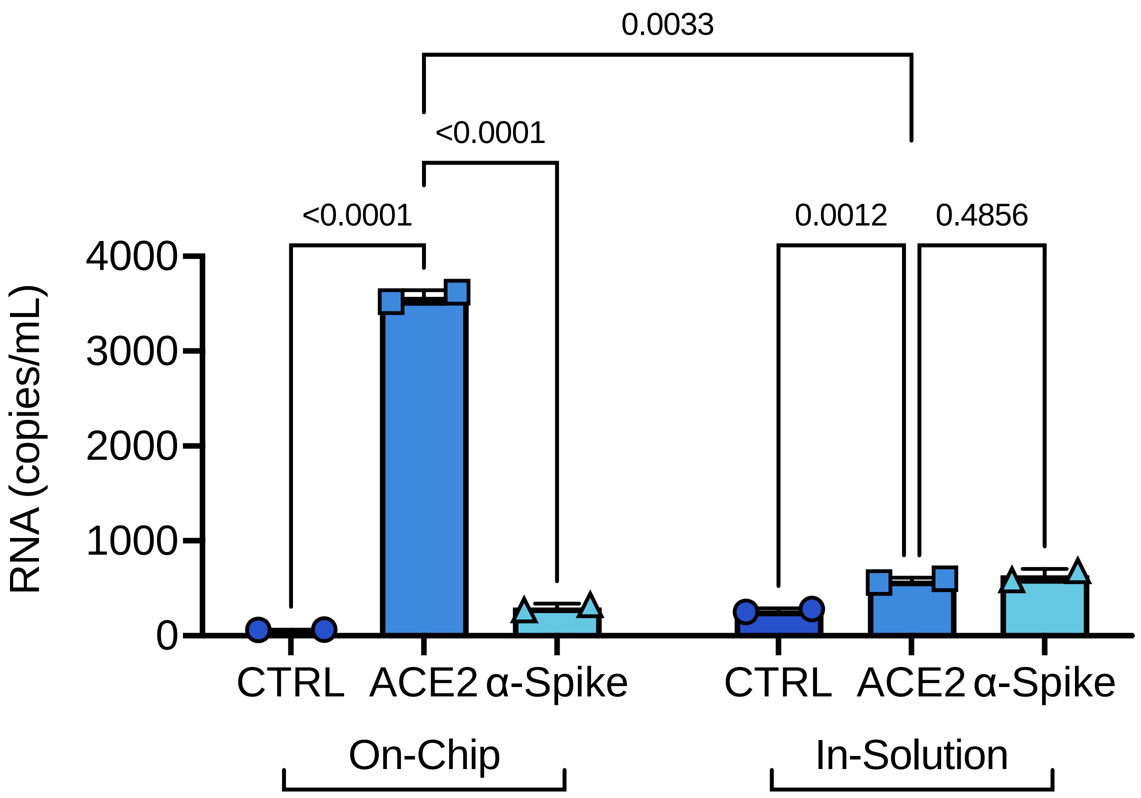

Figure S2: Comparing On-Chip vs In-Solution capture using pseudovirus

SARS-CoV-2 Spike glycoprotein pseudotyped lentivirus (pseudovirus) containing RFP RNA was diluted and spiked into healthy donor plasma. Pseudovirus is captured on the ^{virus}HB-Chip using either a non-specific IgG (CTRL, dark blue circles), ACE2 (light blue squares), or an anti-spike protein antibody (aqua triangles). Capture molecules are added to the device prior to use (left) or incubated with the sample prior to being added to the device (right). RFP RNA was extracted from chips and detected using ddPCR.

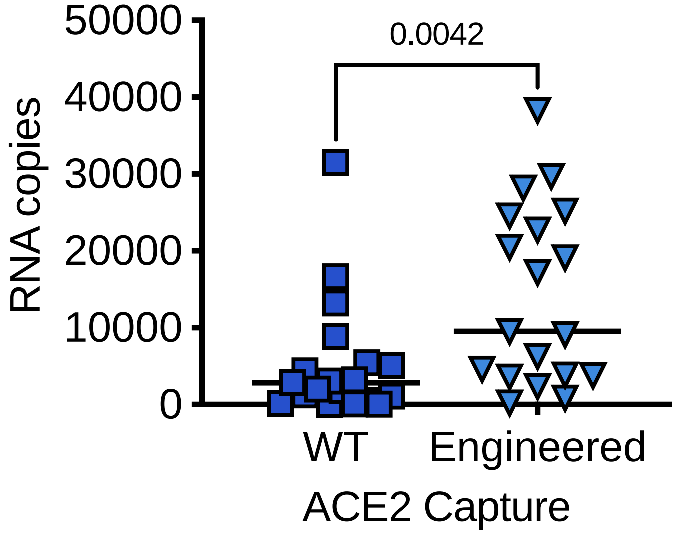

Figure S3: Comparing capture of pseudovirus with Wild Type (WT) or Engineered ACE2

SARS-CoV-2 Spike glycoprotein pseudotyped lentivirus (pseudovirus) containing RFP RNA was diluted and spiked into healthy donor plasma. Pseudovirus was captured on chips either using recombinant wild-type (WT) ACE2 (dark blue squares) or an ACE2 variant engineered for higher binding of spike protein (Engineered, light blue triangles). RFP RNA was extracted from chips and detected using ddPCR.

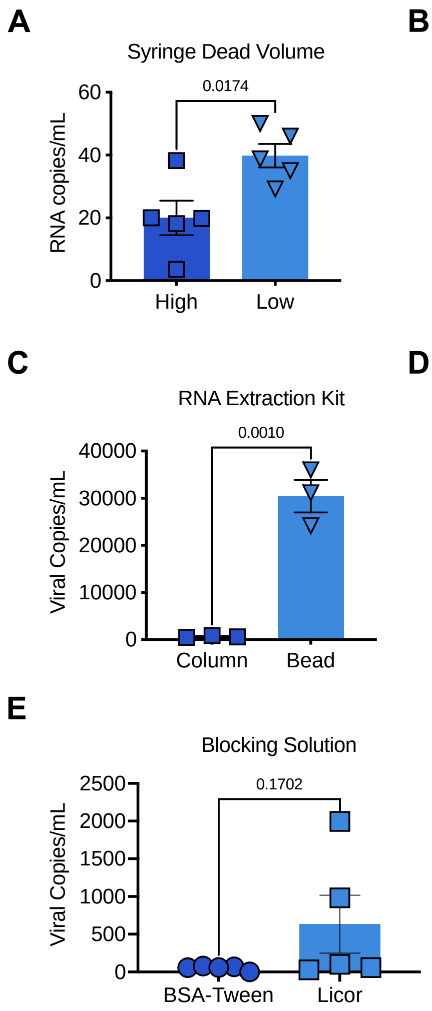

Figure S4: Optimizing the ^{virus}HB-Chip and detection strategy for ultra-sensitive viral detection.

(A-B) Inactivated SARS-CoV-2 was captured on the ^{virus}HB-Chip and detected by ddPCR. **(A)** Viral RNA was detected using reverse transcription followed by ddPCR with SARS-CoV-2 specific probes from IDT (Traditional, blue squares) or one-step RT-ddPCR using the Bio-Rad Triplex SARS-CoV-2 assay (One-Step, light blue triangles). **(B)** RNA was extracted from the ^{virus}HB-Chip using either Direct-zol column-based extraction kits (dark blue squares) or the bead-based MagMAX Viral DNA/RNA pathogen kit (light blue triangles). **(C-D)** SARS-CoV-2 spike protein pseudotyped lentivirus was captured on the ^{virus}HB-Chip and detected by ddPCR. **(C)** Healthy donor plasma spiked with inactivated virus was flown through the ^{virus}HB-Chip using either 1 mL BD syringes (high dead volume, dark blue squares) or Air-Tite syringes (low dead volume, light blue triangles). **(D)** Syringes were loaded on a syringe pump that was either run horizontally (dark blue squares) or vertically pointed down (light blue triangles) through the ^{virus}HB. P-values were calculated using a t-test. **(E)** Prior to SARS-CoV-2 capture, but after capture antibody addition, ^{virus}HB-Chips were blocked with either 3% BSA in PBS containing 0.05% Tween-20 (BSA-Tween, dark blue circles) or with Licor Odyssey Blocking Buffer (Licor, light blue squares).

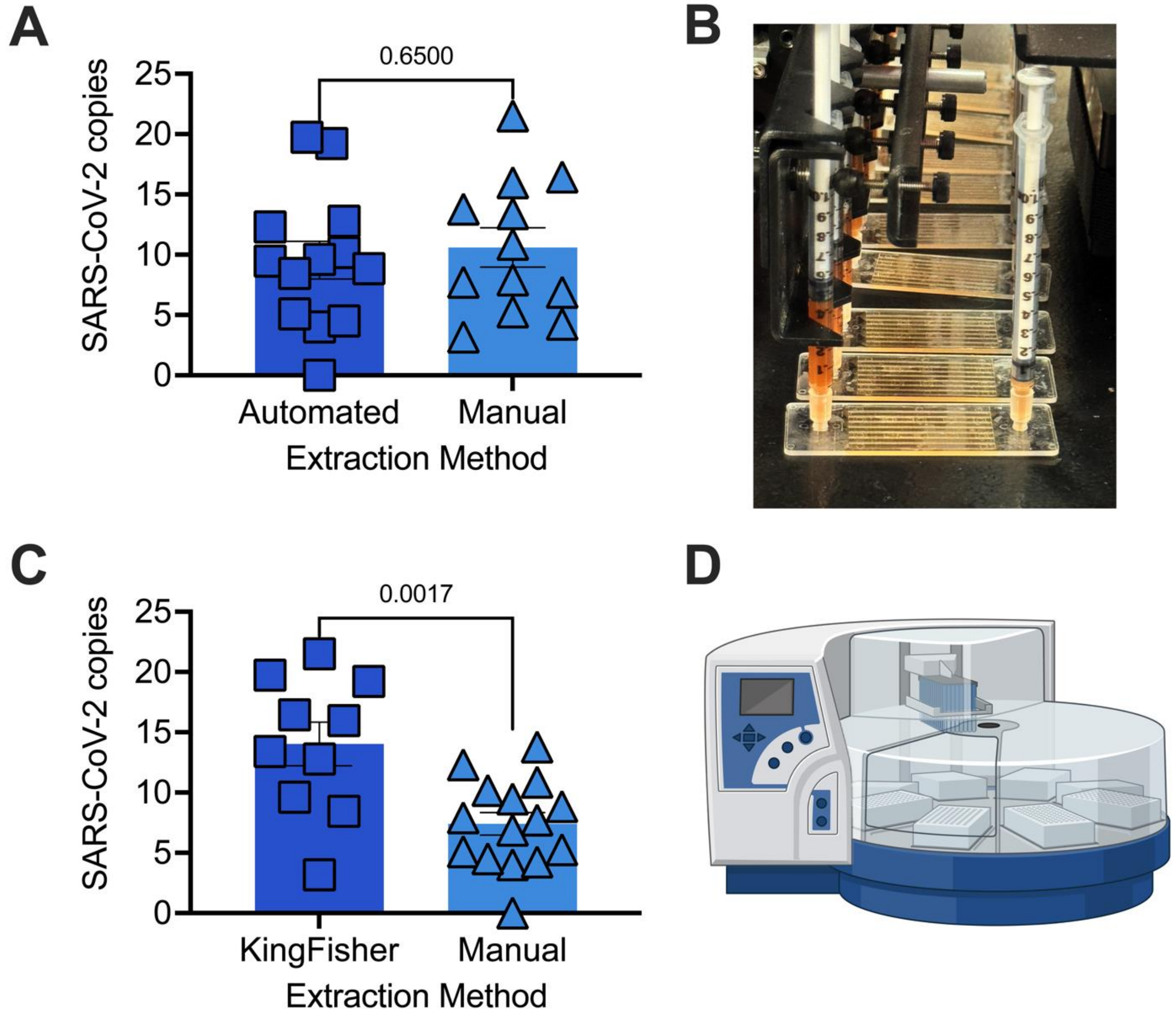

Figure S5: Automation of RNA extraction from the virusHB-Chip.

(A-D) Inactivated SARS-CoV-2 was spiked into healthy donor plasma and then was captured on the virusHB-Chip and detected by one-step RT-ddPCR. **(A)** Samples were extracted using lysis solution from the MagMAX Viral/Pathogen Nucleic Acid Isolation Kit by either manual pushing of solution back and forth through the virusHB-Chip (Manual, light blue triangle) or automated pushing of lysis solution through the virusHB-Chip using syringe pumps (Automated, dark blue squares). **(B)** An image of the automated extraction setup using two syringe pumps. Image taken with an iPhone 15 Pro Max. **(C)** RNA extraction was then performed following either the manufacturer's manual extraction protocol (Manual, light blue triagles) or using the manufacturer's automated extraction protocol on a KingFisher Flex system (KingFisher, dark blue squares). **(D)** A drawing of the KingFisher Flex system showing different plates containing lysate, wash buffers, and elution buffers. Created using BioRender.com.

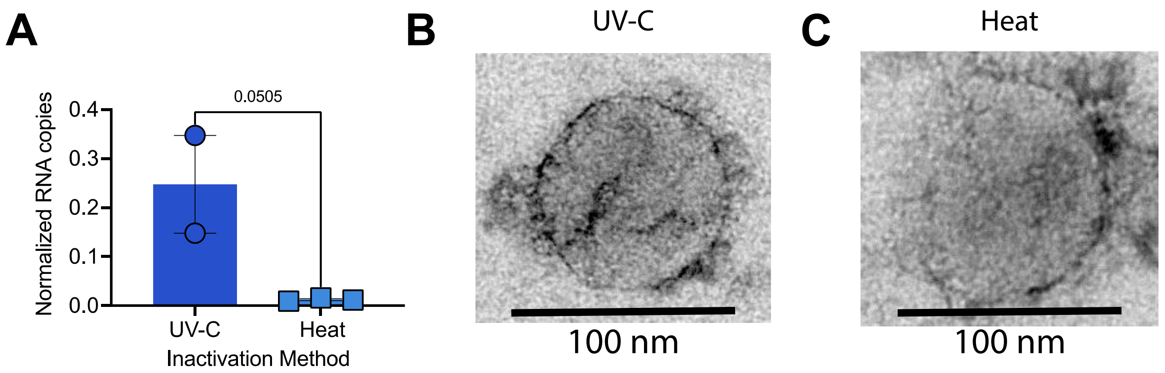

Figure S6: Comparison of UV-C and heat inactivated SARS-CoV-2.

(A) Inactivated SARS-CoV-2 was spiked into healthy donor plasma and then was captured on the ^{virus}HB-Chip and detected by one-step RT-ddPCR. SARS-CoV-2 was inactivated either using UV-C (UV-C, dark blue circles) or 65°C heat treatment (heat, light blue squares). **(B-C)** Representative transmission electron microscopy (TEM) image captured of inactivated WA-1 SARS-CoV-2 that was either inactivated using UV-C **(B)** or 65°C heat treatment **(C)**.

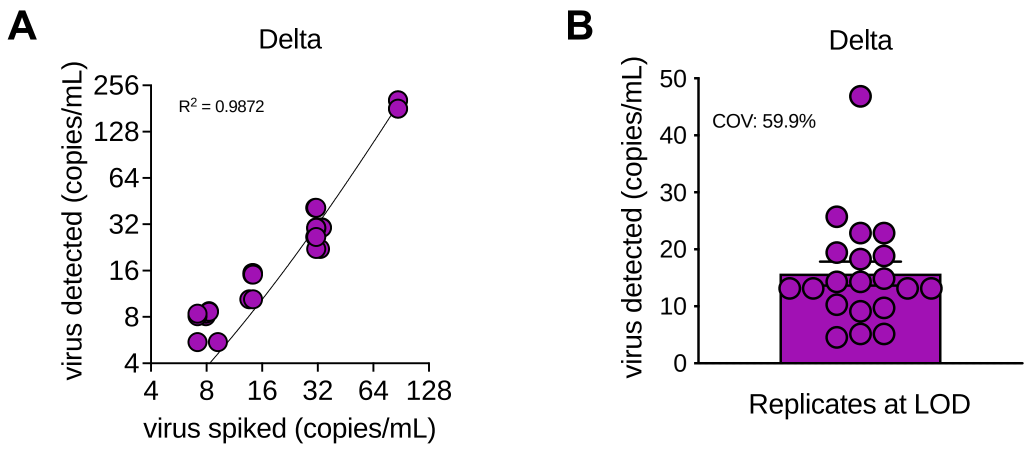

Figure S7: Sensitivity of the ^{virus}HB-Chip.

For all experiments a serial dilution of inactivated SARS-CoV-2 was created and then spiked into healthy donor plasma. One-step RT-ddPCR was performed to detect viral copies captured on chip. **(A)** Copies of Delta detected are graphed on the y-axis versus detected levels of loading controls on the x-axis. **(B)** The same dilution factor as **(A)** was used to prepare Delta at ~8 copies/mL spiked into healthy donor plasma to be captured on the ^{virus}HB-Chip (N=20). A curve fit for dilution series was created using a linear quadratic equation, with R^2 values shown for each.

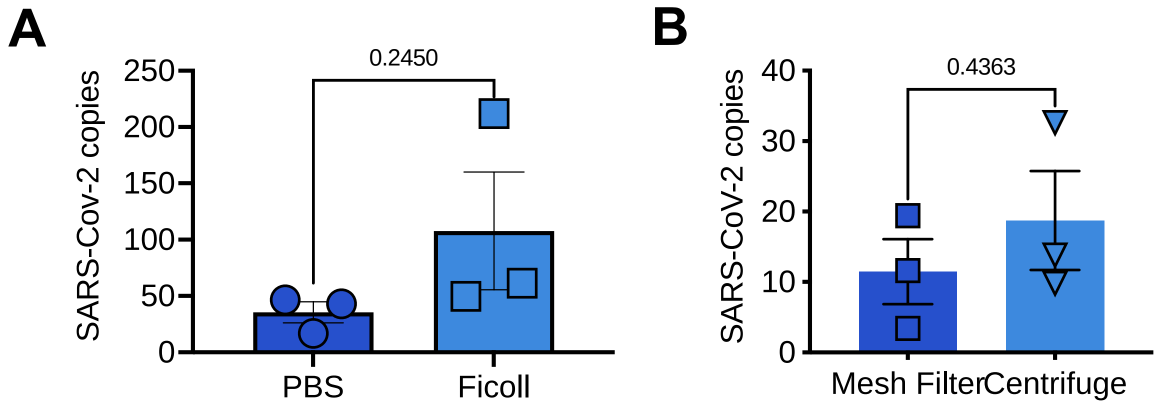

Figure S8: Stool optimization for virusHB-Chip capture.

For all experiments, stool was collected from healthy individuals and spiked with inactivated SARS-CoV-2. Viral RNA was extracted from viral particles captured on chip and the number of bound viral particles was determined using one-step RT-ddPCR. P-values were determined using a T-test. **(A)** Stool samples were diluted in either 2 mL PBS (dark blue circles) or 2 mL Ficoll-PAQUE (light blue squares) per gram of stool prior to being flown through the virusHB-Chip. **(B)** After dilution in Ficoll-PAQUE, samples were either flown through a 40 µm mesh nylon filter (Mesh Filter, dark blue squares) or centrifuged (Centrifuge, light blue triangles) prior to being flown through the virusHB-Chip in order to remove debris and large particulates that would clog our microfluidic device.

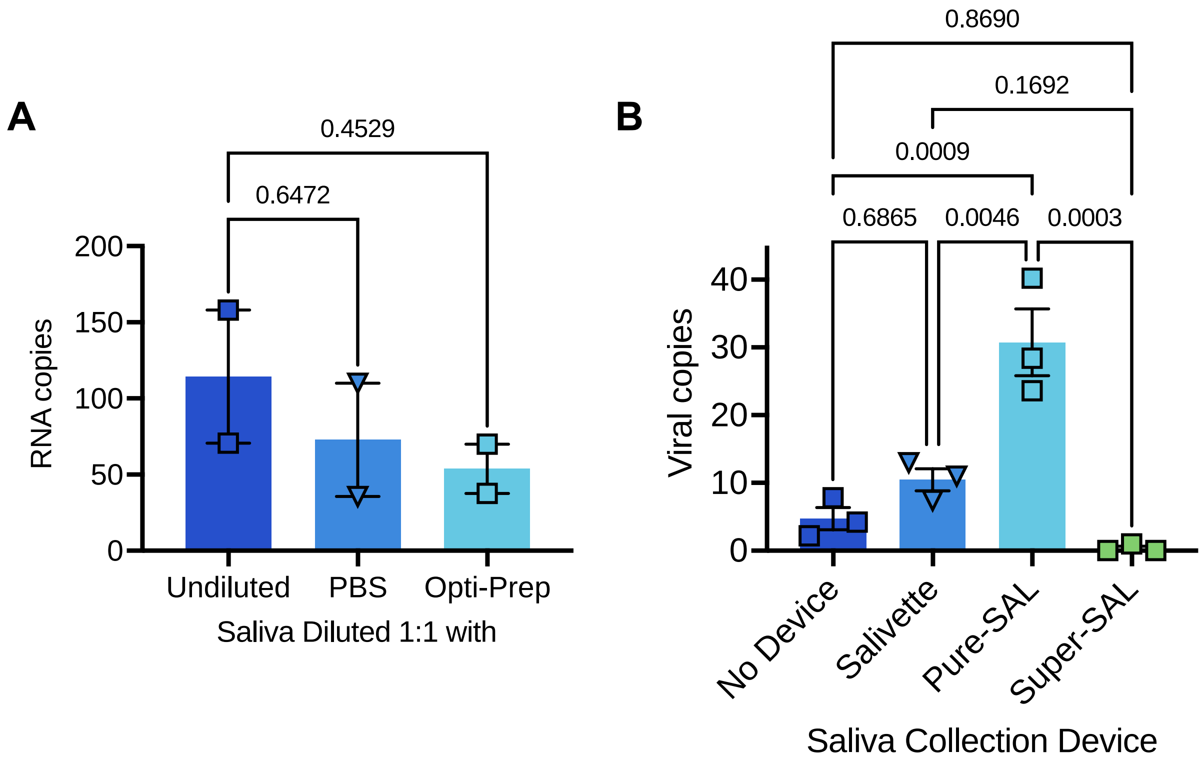

Figure S9: Saliva collection devices for viral detection.

(A) Spike protein pseudotyped lentivirus (pseudovirus) was spiked into saliva from a healthy donor. Spiked saliva was then run undiluted (Undiluted, dark blue squares), diluted 1:1 with 1x PBS (PBS, light blue triangles), or diluted 1:1 with Opti-Prep (Opti-Prep, aqua squares). Spiked samples were then run through the ^{virus}HB-Chip, RNA extracted, and RFP RNA copies determined by ddPCR. Diluted samples were corrected for volume processed when graphed. **(B)** UV-C inactivated SARS-CoV-2 was spiked into healthy donor saliva. RNA was extracted from spiked saliva that was either untreated (No device, dark blue squares), collected with the Salivette (Salivette, light blue triangles), collected with the Pure-SAL (Pure-SAL, aqua squares), or collected with the Super-SAL (Super-SAL, light green squares). RNA was extracted and viral copies were measured using one-step RT-ddPCR. **(A-B)** P-values were obtained using a one-way ANOVA with correction for repeated testing.

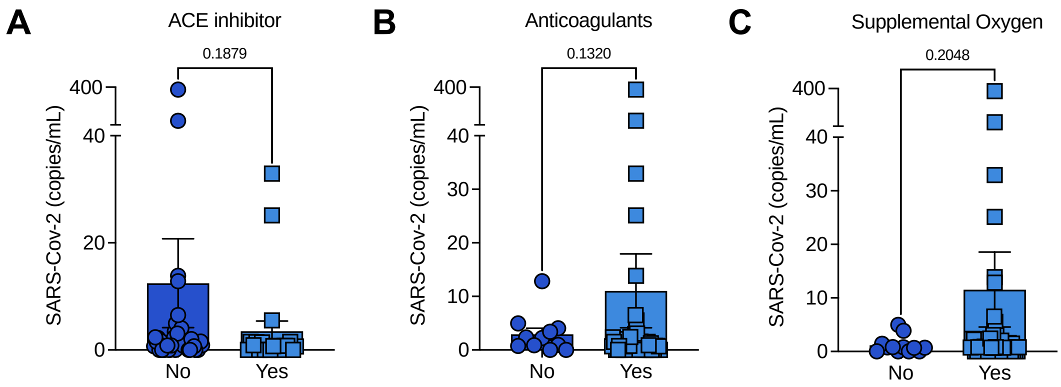

Fig. S10: SARS-CoV-2 levels in plasma in patients receiving different therapies.
Absolute copies/mL of SARS-CoV-2 detected in plasma of COVID-19 patients (N=103) showing differences in levels based on whether they received **(A)** an ACE inhibitor, **(B)** Anticoagulants, or **(C)** Supplemental Oxygen.

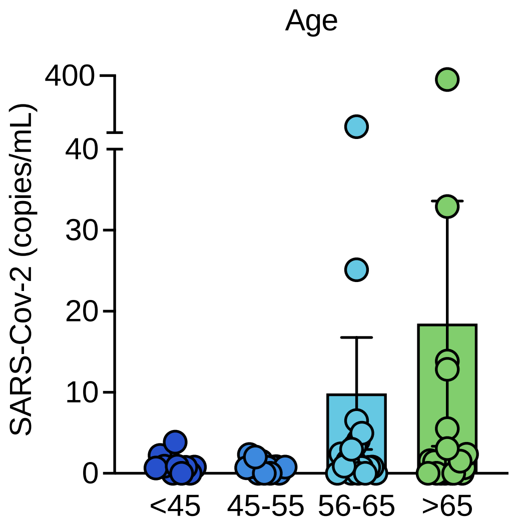

Fig. S11: SARS-CoV-2 levels in plasma in patients of different age group.
Absolute copies/mL of SARS-CoV-2 detected in plasma of COVID-19 patients (N=103) showing differences in levels based on age group of patients.

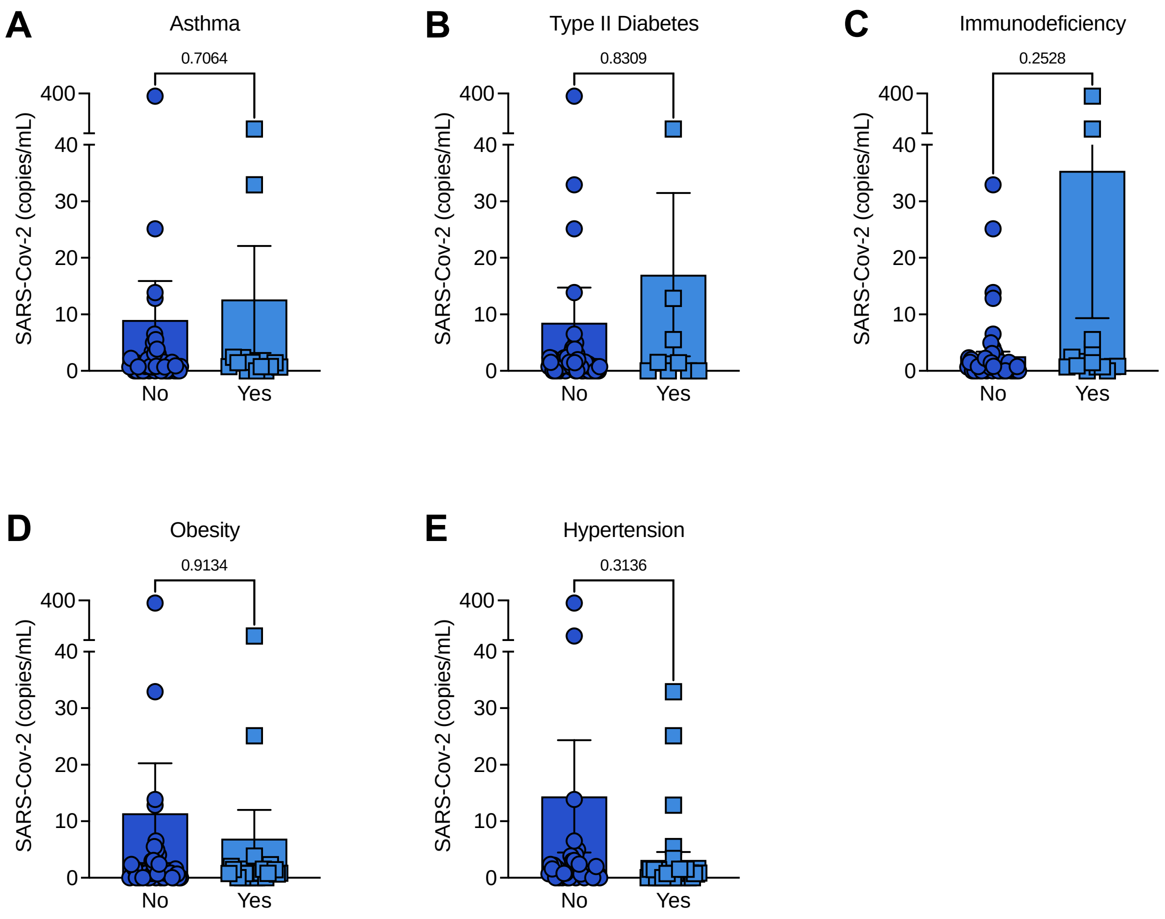

Fig. S12: SARS-CoV-2 levels in plasma in patients with different COVID-19 co-morbidities.

Absolute copies/mL of SARS-CoV-2 detected in plasma of COVID-19 patients (N=103) showing differences in levels based on whether the patient has **(A)** asthma, **(B)** type II diabetes, **(C)** an immunodeficiency, **(D)** obesity, or **(E)** hypertension.

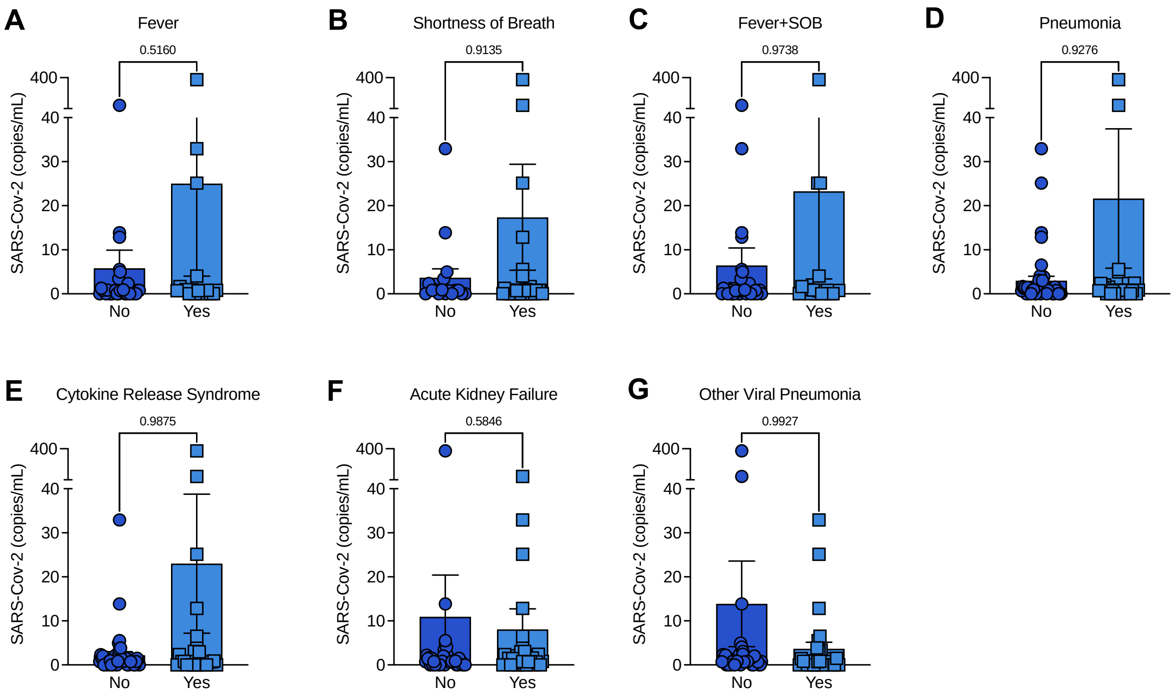

Fig. S13: SARS-CoV-2 levels in plasma of patients with different symptoms.

Absolute copies/mL of SARS-CoV-2 detected in plasma of COVID-19 patients (N=103) showing differences in levels based on whether the patient had the following symptoms or conditions: **(A)** fever, **(B)** shortness of breath, **(C)** fever and shortness of breath, **(D)** pneumonia, **(E)** cytokine release syndrome, **(F)** acute kidney failure, or **(G)** other viral pneumonia.

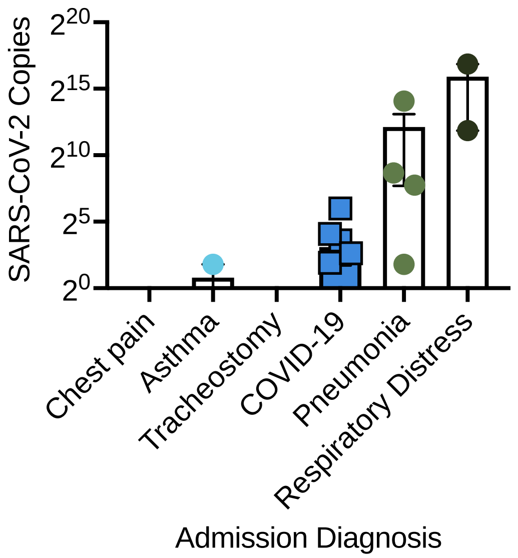

Fig. S14: SARS-CoV-2 levels in saliva of patients with different admission diagnoses.
Absolute copies of SARS-CoV-2 detected in saliva of COVID-19 patients (N=12) depending on the admissions diagnosis from the emergency room.

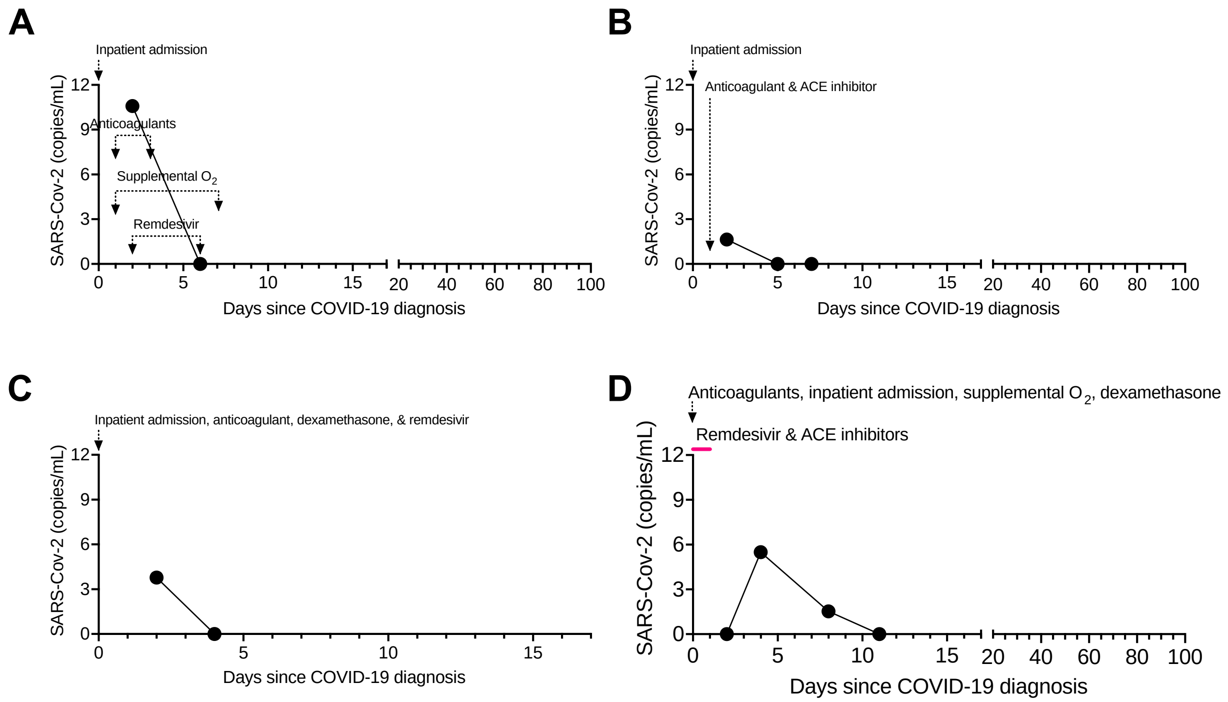

Fig. S15. Serial monitoring of additional samples

(A-D) SARS-CoV-2 copies/mL were detected using the ^{virus}HB-Chip in plasma samples collected over the course of treatment. All patients shown survived and are designated by black circles. These patients displayed few interventions, better outcome, and quick decline in SARS-CoV-2 positivity in plasma. Conditions and treatments are designated by date using colored lines or by arrows for treatments or conditions lasting only one day.

Variant	Inactivation Method	Mode Size (nm)	Concentration		
			particles/mL	Counts ORF1a/mL	Counts N1/mL
WA1	UV-C	75.42	1.27×10^{10}	$9.00x10^{7}$	3.84×10^{8}
WA1	heat	82.50	1.14×10^{10}	$4.90x10^{9}$	1.48×10^{10}
Alpha	UV-C	94.45	8.63×10^{9}	$3.18x10^{8}$	$9.72x10^{8}$
Beta	UV-C	103.54	4.51×10^{9}	$1.12x10^{8}$	$5.54x10^{8}$
Delta	UV-C	88.52	6.18×10^{9}	$1.98x10^{8}$	$5.28x10^{8}$
Omicron (BA.1)	UV-C	84.45	5.33×10^{9}	$6.60x10^{7}$	$2.88x10^{8}$

Table S1. Size and concentration information for inactivated SARS-CoV-2 strains used in this study.

Information table listing the inactivation method, size (mode), and concentrations of each inactivated SARS-CoV-2 strain. Particle/mL concentrations and size (mode) were obtained by nanoparticle tracking analysis. Counts ORF1a and Counts N1 were both obtained by ddPCR.

Patient Demographics					
	**Plasma (n=69)**	**Plasma (n=14)**	**Plasma (n=20)**	**Saliva (n=36)**	**Stool (n=29)**
**Age**					
<45	11	NA	NA	NA	NA
45-55	15	NA	NA	NA	NA
56-65	18	NA	NA	NA	NA
>65	25	NA	NA	NA	NA
**Sex**					
Female	37	NA	NA	15	14
Male	32	NA	NA	21	15
**Race**					
Asian	4	NA	NA	0	2
Black	17	NA	NA	5	4
White	37	NA	NA	18	19
Other	11	NA	NA	8	4
**Ethnicity**					
Non hispanic	50	NA	NA	NA	NA
Hispanic	12	NA	NA	NA	NA
Declined	4	NA	NA	NA	NA
**Collection Date**					
March – June 2020	22	0	20	3	0
June – Oct 2020	7	0	0	2	0
Nov 2020 – Feb 2021	31	4	0	24	29
March – June 2021	6	10	0	0	0
July – Sept 2021	3	0	0	0	0

Table S2. Patient demographic information and collection date ranges for samples. Information table listing the number of samples in each demographic group as well as when during SARS-CoV-2 pandemic samples were collected in Boston, MA. NA = Not Available. For some patient data sets, the numbers do not add up to the total sample number due to unavailable data.

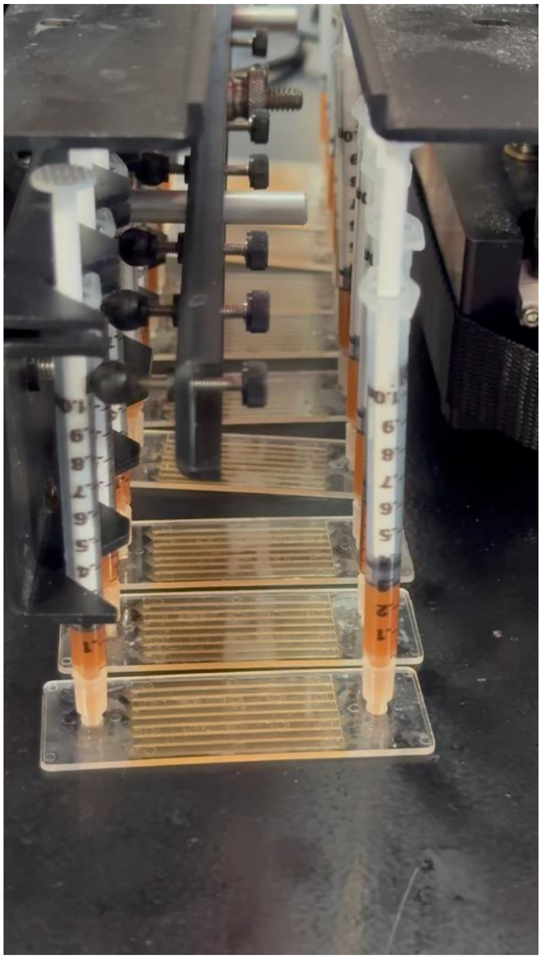

Movie S1.

This movie demonstrates the back and forth pushing between syringe pumps to automate viral extraction of up to 10 chips at a time. 550 µl of lysing solution is added to one syringe and connected to chip inlet. An empty syringe is attached to the chip outlet. The syringe pump is programmed to push 550 µl through the chips, then delay 5 seconds to account for slow flow due to high viscosity of lysing solution. The syringe pump then withdraws 550 µl allowing for the opposing syringe pump to push the lysing solution back through, followed by a 5 second delay. This process is repeated for a total of 8 times. Because the opposing syringe pump is operated in the opposite direction, an identical program to the first syringe pump is used.
